# Optimizing adjuvant strategies for sevoflurane-related emergence delirium: a Bayesian network meta-analysis in pediatric surgery

**DOI:** 10.3389/fphar.2025.1573640

**Published:** 2025-07-04

**Authors:** Chun-Jin Zhang, Hong Chen, Kang Zou, Xi Qu

**Affiliations:** ^1^ MSN, RN, Fellow,Nursing department, Tongji Hospital, Tongji Medical College, Huazhong University of Science and Technology, Wuhan, Hubei, China; ^2^ MSN, RN, Chief Head Nurse, Nursing department, Tongji Hospital, Tongji Medical College, Huazhong University of Science and Technology, Wuhan, Hubei, China; ^3^ MSN, RN, Teaching Supervision,Nursing department, Tongji Hospital, Tongji Medical College, Huazhong University of Science and Technology, Wuhan, Hubei, China

**Keywords:** emergence delirium, pediatric, adjuvant, sevoflurane-related, network meta-analysis

## Abstract

**Objective:**

To compare the efficacy of different anesthetic adjuvants combined with sevoflurane across specific surgical sites using a Bayesian network meta-analysis.

**Methods:**

A systematic review was conducted, following PRISMA guidelines, including 100 randomized controlled trials (RCTs) involving 8,800 pediatric patients undergoing various surgeries. The network meta-analysis evaluated 22 drug interventions, with log risk ratios (logRR) and Surface Under the Cumulative Ranking (SUCRA) probabilities calculated for each drug or combination.

**Results:**

Among all interventions, dexmedetomidine combined with alfentanil was the most effective in reducing ED risk for tonsillectomy/adenoidectomy, achieving a SUCRA ranking of 94.63% (logRR = −2.82). For ophthalmic surgery, propofol and midazolam showed the highest efficacy (logRR = −1.83, SUCRA: 86.03%). Dexmedetomidine combined with midazolam was the optimal combination for inguinal hernia/hypospadias (logRR = −2.16, SUCRA: 81.73%) and dental/oral repairs (logRR = −1.83, SUCRA: 94.85%). For cleft lip/palate repair, dexmedetomidine alone showed significant efficacy (logRR = −1.65, SUCRA: 89.15%). In myringotomy/cochlear implantation, fentanyl was the most effective adjuvant (logRR = −1.17, SUCRA: 80.02%).

**Conclusion:**

Targeted use of dexmedetomidine-based combinations was found to be particularly effective across various surgeries, while fentanyl and propofol-midazolam combinations excelled in specific contexts. This study underscores the importance of tailoring anesthetic adjuvant strategies to specific surgical sites to reduce the risk of ED in pediatric patients, and provides a valuable reference for optimizing anesthetic care in this vulnerable population.

## 1 Introduction

Sevoflurane is the most commonly used drug for induction and maintenance of anesthesia in pediatric patients (Sakai et al., 2005). It provides rapid induction and recovery, with easy adjustment of anesthetic depth. Additionally, sevoflurane has minimal impact on heart rate and little airway irritation, allowing for muscle relaxation. However, its use is associated with a high incidence of postoperative emergence delirium (ED) in the pediatric population (Park et al., 2014; Dahmani et al., 2010; Kulka et al., 2001), potentially leading to self-injury, delayed discharge, and increased medical costs (Park et al., 2014; Dahmani et al., 2010; Kulka et al., 2001).

Anesthetic adjuvants such as dexmedetomidine, ketamine, propofol, fentanyl, midazolam, sufentanil, remifentanil, and clonidine are effective in preventing ED (Fang et al., 2016). A network meta-analysis (NMA) demonstrated that dexmedetomidine combined with sevoflurane appeared to be the most effective strategy for reducing the risk of ED in pediatric anesthesia, compared to other single adjuvant agents (Wang et al., 2017). Previous randomized controlled trials (RCTs) and meta-analyses have also shown that combination therapies may have a synergistic effect in preventing ED. Specifically, the combination of dexmedetomidine, midazolam, and an antiemetic was identified as the most effective strategy to prevent ED (Wang et al., 2021). However, these meta-analyses did not differentiate between various surgical sites and types, treating all pediatric surgical populations as a single homogeneous group, which may introduce bias. Previous meta-analyses have indicated that head and neck surgeries are associated with a higher incidence of pediatric ED (Chen et al., 2024). Furthermore, ophthalmic and ear, nose, and throat (ENT) surgeries are considered risk factors for ED (Dahmani et al., 2010). Children undergoing ophthalmic and ENT surgeries often experience anxiety related to visual impairment, a sense of choking from restricted speech, or discomfort with swallowing, which may increase the risk of ED (Joo et al., 2014; Eckenhoff et al., 1961). In cleft palate repairs, anatomical reconstruction of the soft and hard palates may result in significant postoperative oropharyngeal pain and bleeding (Liu et al., 2021), and pain is a major risk factor for ED. Inadequate pain management may lead to delirium. For pediatric inguinal hernia repair, the incidence of ED significantly decreases when sevoflurane anesthesia is combined with caudal block (Aouad et al., 2005).

Currently, meta-analyses comparing the efficacy of different anesthetic adjuvants for preventing ED in specific surgical sites are limited to three studies: one focusing on ophthalmic surgery (Tan et al., 2019), another on tonsillectomy/adenoidectomy (Jiao et al., 2020), and the third on cleft palate surgery (Liu et al., 2021). However, the first two studies did not comprehensively compare the combined effects of different anesthetic adjuvants, and the last one only analyzed the effect of dexmedetomidine. To compare the efficacy of combination or single therapies in preventing ED across different surgical sites, we used a Bayesian network to identify which anesthetic adjuvants combined with sevoflurane influence the incidence of ED in pediatric surgical patients and to determine the best strategy for guiding anesthetic practice.

## 2 Methods

### 2.1 Study strategy and selection criteria

This NMA follows the PRISMA (Preferred Reporting Items for Systematic Reviews and Meta-Analyses) 2020 guidelines and is registered in PROSPERO. A systematic review was conducted of publications retrieved from PubMed, Embase, Cochrane Library, and ClinicalTrials.gov databases, covering records up until 29 September 2024. The search terms used included: “ (anesthesia) and (sevoflurane) and [(delirium) or (agitation)] and [(ancillary drug) or (ketamine) or (propofol) or (dexmedetomidine) or (clonidine) or (midazolam) or (fentanyl) or (remifentanil) or (sufentanil) or (melatonin)],” along with their synonyms. No restrictions were placed on language or publication date. For non-English articles, we used Google Translate for translation. Additionally, we manually searched reference lists of review articles and pairwise meta-analyses for potentially eligible studies. As this study is a systematic review and meta-analysis, no new human or animal data were collected. Ethical approval was not required. All included RCTs had previously obtained ethical approval from their respective institutions.

### 2.2 Inclusion and exclusion criteria

The PICO criteria applied for this NMA are as follows: (1) Patients or Population: Pediatric patients (under 18 years old) undergoing general anesthesia with sevoflurane, with specific surgical sites reported; (2) Intervention: Drug interventions administered during sevoflurane general anesthesia; (3) Control: Placebo control or active control groups; (4) Outcome: Incidence of ED following sevoflurane anesthesia.

Exclusion criteria included: (1) Studies that were not randomized controlled trials (RCTs), (2) Studies not reporting ED incidence, (3) Studies unrelated to drug interventions targeting the risk of ED, or (4) Studies involving patients not receiving sevoflurane anesthesia. In cases of duplicate data, only the report with more information and a larger sample size was included.

### 2.3 Data extraction

Titles and abstracts identified through the database search were exported to end note X9, with duplicates removed. Two researchers independently screened the titles and abstracts for eligibility, followed by full-text reviews of potentially relevant studies to determine final inclusion. Discrepancies during the selection process were resolved by a third reviewer. From each included study, we extracted the following information: first author, year of publication, type of surgery, type of anesthetic adjuvants, patient demographics, sample size, and the number of ED cases.

### 2.4 Quality assessment

The quality of the included studies was assessed using the Cochrane Risk of Bias tool, which evaluates the following domains: random sequence generation (selection bias), allocation concealment (selection bias), blinding of participants and personnel (performance bias), blinding of outcome assessment (detection bias), incomplete outcome data (attrition bias), selective reporting (reporting bias), and other potential sources of bias.

### 2.5 Statistical analysis

Data were analyzed using R version 4.3.3, and network evidence along with comparison-adjusted funnel plots were generated using Stata 17. For categorical outcomes, log risk ratios (logRRs) and 95% credible intervals (CrIs) were calculated. The NMA was conducted within a Bayesian framework using Markov Chain Monte Carlo (MCMC) simulation. Leverage plots were used to evaluate model convergence (Supplementary Figure S1). The Bayesian approach was chosen over frequentist methods due to its flexibility in incorporating prior information and fully probabilistic interpretation of treatment effects and rankings. Unlike traditional meta-analytic techniques, which provide point estimates and confidence intervals, the Bayesian framework allows direct probability statements about treatment rankings and uncertainty. This is particularly valuable in NMA, where indirect and mixed comparisons are synthesized simultaneously (Béliveau et al., 2019).

To aid the interpretation of treatment rankings, Surface Under the Cumulative Ranking (SUCRA) values were calculated. SUCRA represents the percentage of efficacy/safety that an intervention achieves relative to a hypothetical ideal treatment that is always the best. A SUCRA of 1 (or 100%) indicates the treatment is most likely to be the best, whereas 0 indicates the least effective, which can support clinical decision-making by offering a comparative measure of benefit across multiple treatments (Mbuagbaw et al., 2017).

For the sensitivity analysis, meta-regression analyses were also conducted within a Bayesian framework to investigate the influence of confounding factors, including participants’ mean age (continuous variable), proportion of males (continuous variable), time of prescription (before or at the start of anesthesia and surgery = 1, during anesthesia in surgery = 2, near the end or at the end of surgery = 3), and risk of bias in studies (low = 1, some concerns = 2, high = 3). These models employed the MCMC method with 10,000 burn-in iterations and an additional 500,00 simulations, utilizing four chains with different initial values to derive medians and 95% credible intervals (CrIs) (Neupane et al., 2014). Comparison-adjusted funnel plots were employed to assess the presence of small-study effects.

## 3 Results

### 3.1 Study characteristics

**FIGURE 1:**
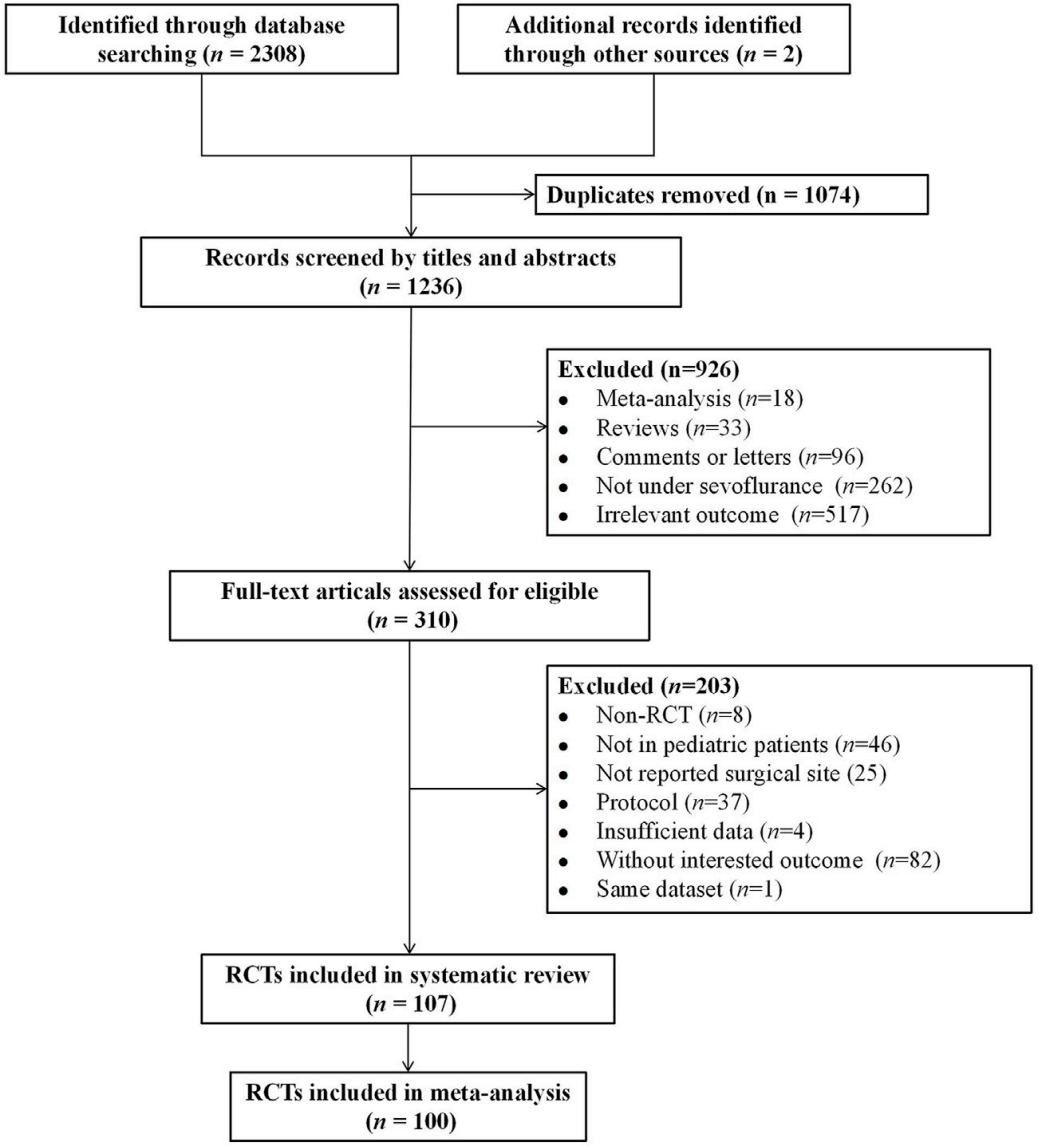
Flowchart of the study selection.

A total of 2,310 records were identified through the literature search. Of these, 1,074 were duplicate articles. After reviewing the titles and abstracts, 926 unrelated articles were excluded, and an additional 203 articles were excluded for not meeting the inclusion criteria. 107 eligible articles were included in the systematic review. Among them, seven studies investigating surgical types that involved non-invasive and painless MRI procedures were excluded (Abu-Shahwan, 2008; Bong et al., 2015; Costi et al., 2015; Cravero et al., 2003; Dalens et al., 2006; Isik et al., 2006; Moawad and El-Diasty, 2015). Ultimately, 100 studies were included in the final NMA (Abbas et al., 2019; Abdelaziz et al., 2016; Abdelhalim and Alarfaj, 2013; Abdelmawgoud and Mohy, 2012; Abu-Shahwan and Chowdary, 2007; Akin et al., 2012; Alansary et al., 2023; Ali and Abdellatif, 2013; Ali et al., 2020; Almenrader et al., 2007; Amer et al., 2022; Amer et al., 2022; Aouad et al., 2007; Asaad et al., 2011; Bae et al., 2010; Baek et al., 2022; Bakhame et al., 2009; Bedirli et al., 2017; Bergendahl et al., 2004; Bilgen et al., 2014; Bromfalk et al., 2023; Cai et al., 2021; Cai et al., 2021; Cai et al., 2024; Chen et al., 2018; Chen et al., 2010; Chen et al., 2013; Chen et al., 2023; Cho et al., 2014; Choi et al., 2016; Demirbilek et al., 2004; Di et al., 2017; Di et al., 2014; Di et al., 2014; Dong et al., 2010; Erdil et al., 2009; Finkel et al., 2001; Galinkin et al., 2000; Ghai et al., 2010; Ghosh et al., 2011; Golmohammadi et al., 2024; Guler et al., 2005; Hadi et al., 2015; Hauber et al., 2015; Hauber et al., 2015; He et al., 2023; Heinmiller et al., 2013; Huang et al., 2022; Ibacache et al., 2004; Ibrahim et al., 2023; Jangra et al., 2022; Jayaraj et al., 2023; Jeong et al., 2012; Ju et al., 2013; Jun et al., 2018; Jun et al., 2018; Jung et al., 2010; Kawai et al., 2019; Khalifa and Hassanin, 2013; Kim et al., 2009; Kim et al., 2016; Kim et al., 2013; Kim et al., 2014; Kim et al., 2011; Komazaki et al., 2020; Lankinen et al., 2006; Lankinen et al., 2006; Lee C. J. et al., 2010; Lee Y. S. et al., 2010; Li et al., 2011; Li et al., 2013; Li et al., 2024; Liang et al., 2014; Lili et al., 2012; Lin et al., 2017; Lin et al., 2016; Liu et al., 2022; Liu et al., 2022; Lundblad et al., 2015; Luo et al., 2017; Meng et al., 2012; Mizrak et al., 2011; Mizrak et al., 2010; Mohamed Maaly et al., 2024; Na et al., 2013; Patel et al., 2010; Peng and Zhang, 2015; Pestieau et al., 2011; Pestieau et al., 2011; Rashad and Soud, 2014; Saadawy et al., 2009; Sahmeddini et al., 2024; Shen et al., 2012; Sheta et al., 2014; Shi et al., 2019; Soliman and Alshehri, 2015; Song et al., 2016; Sousa-Júnior et al., 2021; Thomas et al., 2015; Thomas et al., 2015; Vettuvanthodi et al., 2024; Viitanen et al., 1999; Xi et al., 2012; Xiao et al., 2012; Xing et al., 2024; Yang et al., 2022; Yao et al., 2015; Yun et al., 2016; Zhang et al., 2022). The selection process is illustrated in Figure 1. The basic characteristics of the included studies are presented in Supplementary Table S1. In total, 8,800 children with varying health conditions were covered in the studies. Twenty-three studies were three-arm trials, while the rest were two-arm studies.

### 3.2 Risk of bias assessment

**FIGURE 2:**
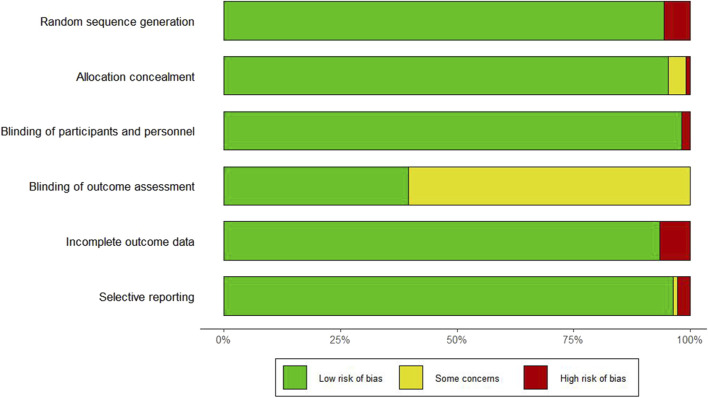
Risk of bias graph and evaluators.

The risk of bias assessment for the seven domains across the 106 studies is provided in Supplementary Table S2 and Figure 2. Six studies did not report the use of randomization methods, one study did not specify allocation concealment, and two studies did not apply blinding for outcome assessment.

### 3.3 Outcomes

#### 3.3.1 Overall surgical sites

**FIGURE 3:**
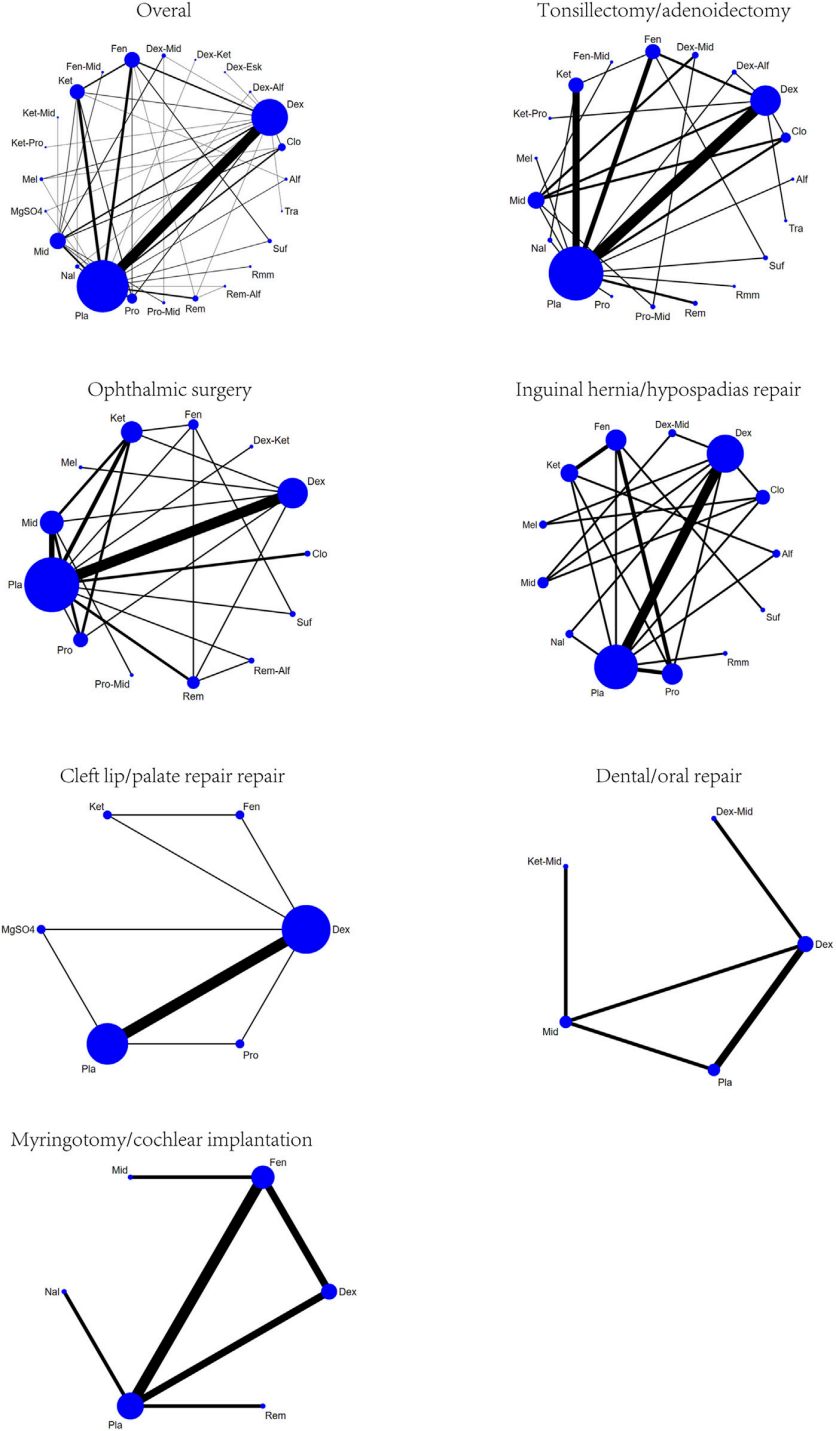
Network plots for different surgical sites.

**FIGURE 4:**
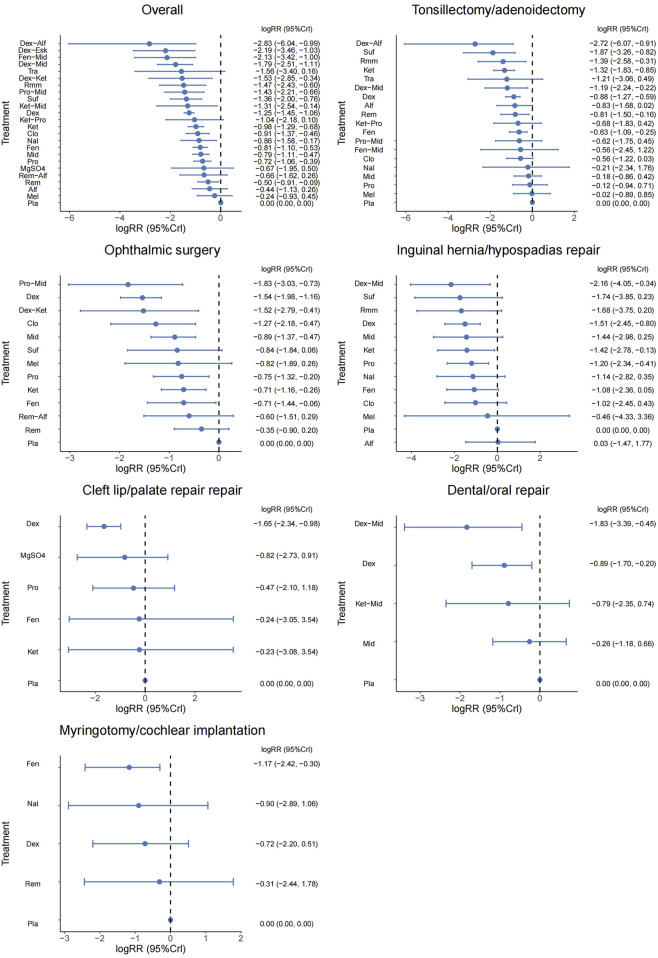
Forest plot presenting the results for all interventions directly compared with the placebo group.

**FIGURE 5:**
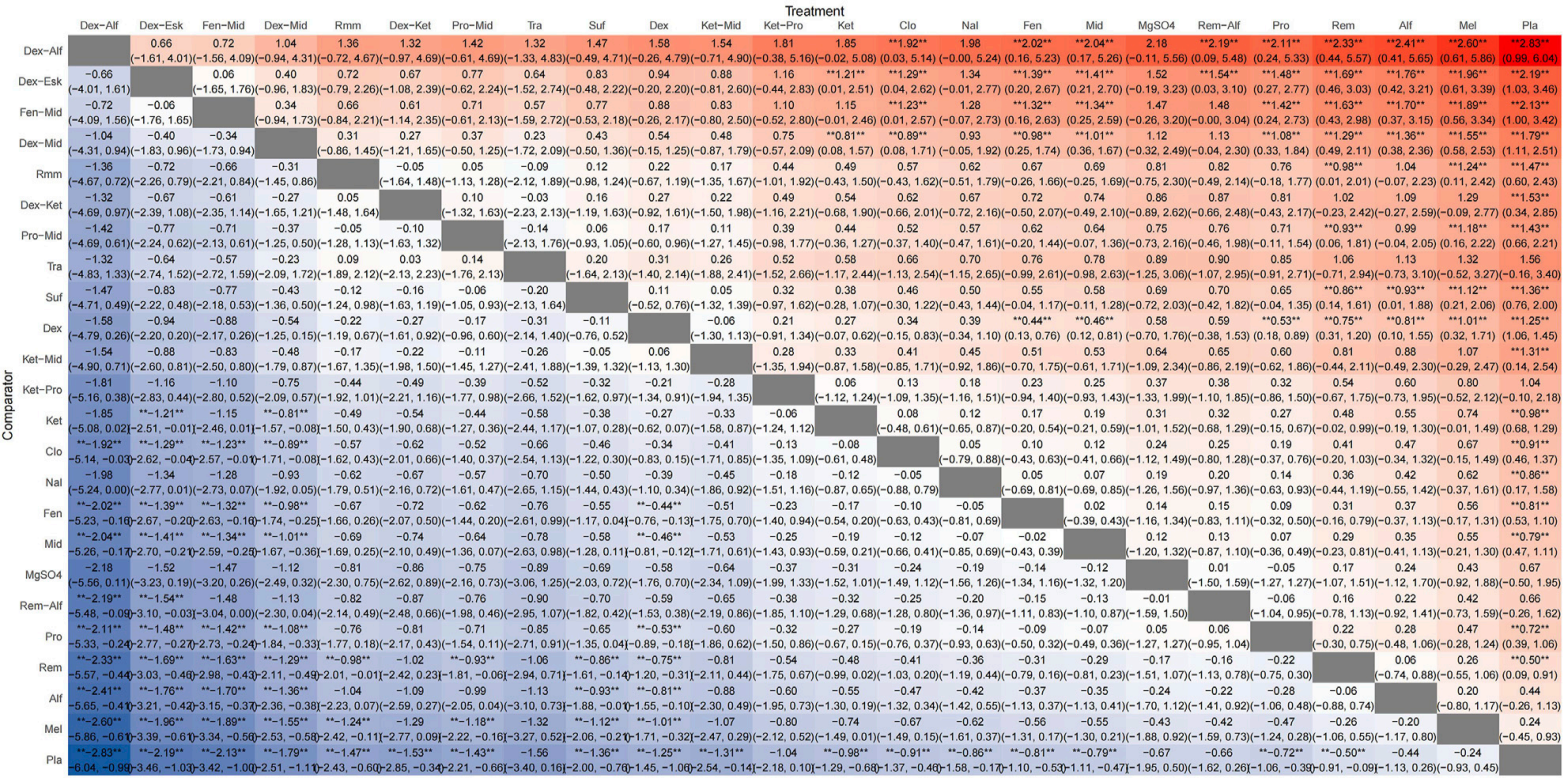
League plots for anesthetic adjuvants in all surgical sites.

**FIGURE 6:**
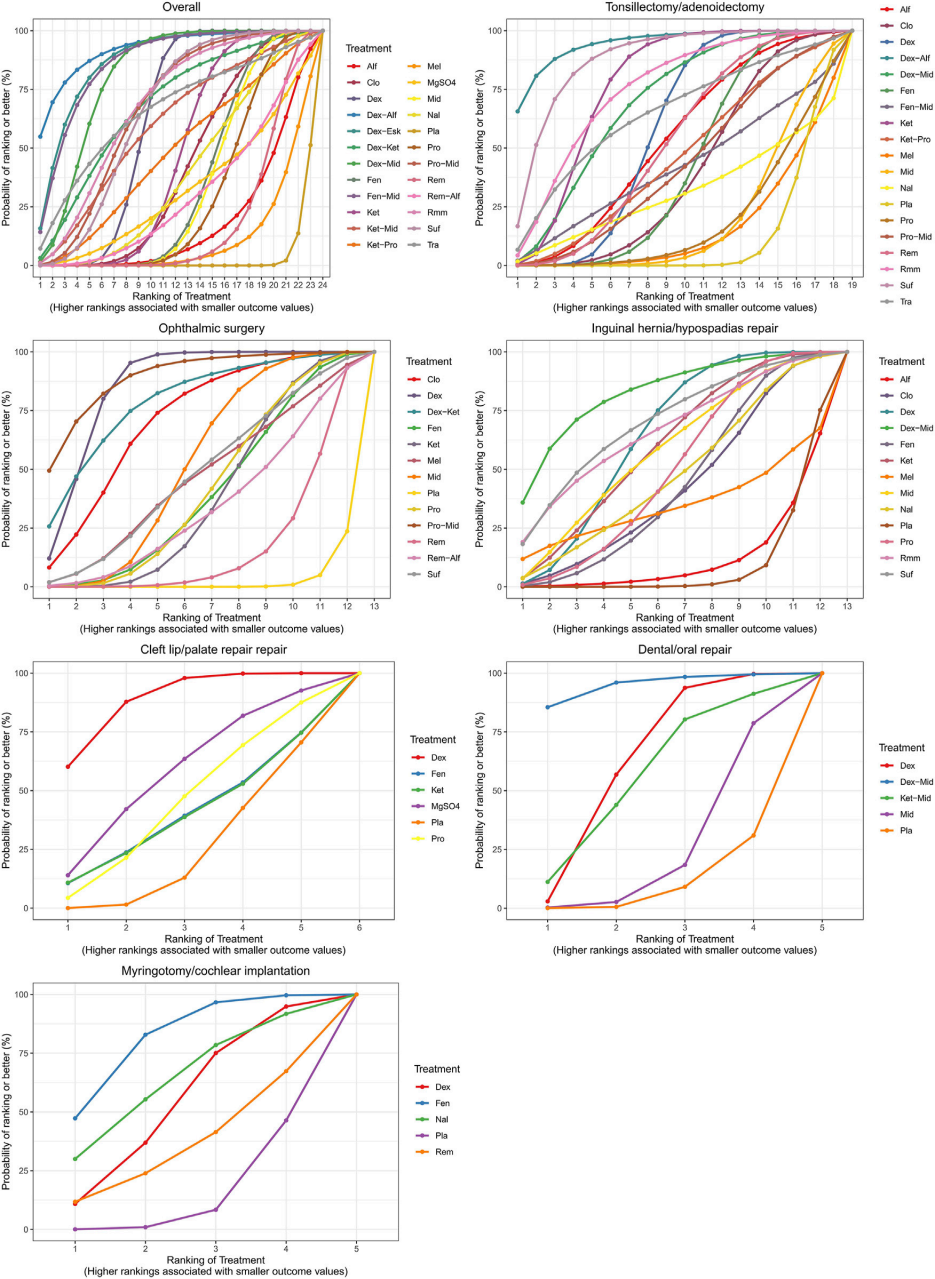
Surface under the cumulative ranking curve plots for different surgical sites.

A total of 100 studies were included, covering 24 different drugs or drug combinations. The network evidence diagram is presented in Figure 3. Compared to placebo, most drug interventions were significantly associated with a reduced incidence of ED (Figure 4). The top three most effective single drugs or combinations were dexmedetomidine + alfentanil (logRR = −2.83, 95% CrI: −6.04, −0.99; SUCRA: 91.7%), dexmedetomidine + esketamine (logRR = −2.19, 95% CrI: −3.46, −1.03; SUCRA: 85.9%), and fentanyl + midazolam (logRR = −2.13, 95% CrI: −3.42, −1.00; SUCRA: 82.8%) (Figures 5, 6).

#### 3.3.2 Tonsillectomy/adenoidectomy

A total of 35 studies involving 19 different drugs or drug combinations and 3,238 children were included. The network evidence diagram is shown in Figure 3. Compared to placebo/control, the top three most effective drugs or combinations were dexmedetomidine + alfentanil (logRR = −2.72, 95% CrI: −6.07, −0.91; SUCRA: 94.63%), sufentanil (logRR = −1.87, 95% CrI: −3.26, −0.82; SUCRA: 87.84%), and remimazolam (logRR = −1.39, 95% CrI: −2.58, −0.31; SUCRA: 76.62%) (Figures 4, 6 and Supplementary Figure S2).

#### 3.3.3 Ophthalmic surgery

A total of 26 studies involving 13 different drugs or drug combinations and 2,019 children were included. The network evidence diagram is shown in Figure 3. Compared to placebo/control, the top three most effective drugs or combinations were propofol + midazolam (logRR = −1.83, 95% CrI: −3.03, −0.73; SUCRA: 89.6%), dexmedetomidine (logRR = −1.54, 95% CrI: −1.98, −1.16; SUCRA: 86.0%), and dexmedetomidine + ketamine (logRR = −1.52, 95% CrI: −2.79, −0.41; SUCRA: 79.5%) (Figures 4, 6 and Supplementary Figure S3).

#### 3.3.4 Inguinal hernia/hypospadias repair

A total of 17 studies involving 13 different drugs or drug combinations and 1,576 children were included. The network evidence diagram is shown in Figure 3. The most effective treatments compared to control were dexmedetomidine + midazolam (logRR = −2.16, 95% CrI: −4.05, −0.34; SUCRA: 81.7%), dexmedetomidine (logRR = −1.51, 95% CrI: −2.45, −0.80; SUCRA: 62.4%) and ketamine (logRR = −1.42, 95% CrI: −2.78, −0.13; SUCRA: 57.4%) (Figures 4, 6 and Supplementary Figure S4).

#### 3.3.5 Cleft lip/palate repair

A total of 10 studies involving 6 different drugs or drug combinations and 541 children were included. The network evidence diagram is shown in Figure 3. Compared to the control group, only dexmedetomidine (logRR = −1.65, 95% CrI: −2.34, −0.98; SUCRA: 89.2%) showed a statistically significant reduction in the incidence of ED (Figures 4, 6 and Supplementary Figure S5).

#### 3.3.6 Dental/oral repair

A total of 6 studies involving 5 different drugs or drug combinations and 450 children were included. The network evidence diagram is shown in Figure 3. Dexmedetomidine + midazolam (logRR = −1.83, 95% CrI: −3.39, −0.45; SUCRA: 94.9%) and dexmedetomidine (logRR = −0.89, 95% CrI: −1.70, −0.20; SUCRA: 63.3%) were associated with a significant reduction in the incidence of ED (Figures 4, 6 and Supplementary Figure S6).

#### 3.3.7 Myringotomy/cochlear implantation

A total of 6 studies involving 6 different drugs or drug combinations and 576 children were included. The network evidence diagram is shown in Figure 3. Fentanyl (logRR = −1.17, 95% CrI: −2.42, −0.30; SUCRA: 80.02%) showed significantly better effects compared to placebo (Figures 4, 6 and Supplementary Figure S7).

### 3.4 Network meta-regression and publication bias

Results of network meta-regression found that, among the various surgical types, the Tonsillectomy/adenoidectomy group showed mean age potentially influences the association between anesthetic adjuvants and sevoflurane-related ED (β = −0.470, 95% CrI: −0.921, −0.031), with a 95% credible interval that does not include zero (Table 1). However, in all other surgical subgroups, none of the analyzed variables demonstrated statistical significance, further supporting the robustness of our main findings, which remain unaffected by these confounding factors.

**TABLE 1:** Results of network meta-regression.

Surgical sites	Meta regression β (95% CrI)			
	Mean age (year)	Male (%)	Time of prescription	Risk of bias
Overall surgical sites	−0.244 (−0.536, 0.034)	0.08 (−0.202, 0.388)	−0.056 (−0.36, 0.256)	0.044 (−0.223, 0.325)
Tonsillectomy/adenoidectomy	**−0.47 (-0.921, -0.031)**	0.202 (−0.206, 0.657)	0.017 (−0.499, 0.608)	0.062 (−0.4, 0.527)
Ophthalmic surgery	0.139 (−0.423, 0.771)	0.173 (−0.416, 0.826)	−0.362 (−1.209, 0.323)	−0.13 (−0.864, 0.524)
Inguinal hernia/hypospadias repair	−0.113 (−1.722, 1.528)	0.093 (−2.799, 3.693)	0.419 (−1.473, 2.52)	0.424 (−1.075, 2.29)
Cleft lip/palate repair	−0.707 (−3.581, 2.255)	−0.84 (−7.758, 2.993)	0.137 (−1.355, 1.579)	−0.25 (−2.744, 1.279)
Dental/oral repair	−0.097 (−1.504, 1.284)	−0.361 (−2.032, 0.982)	−0.431 (−1.896, 0.935)	−0.158 (−1.921, 1.47)
Myringotomy/cochlear implantation	0.114 (−1.648, 1.923)	0.03 (−1.999, 2.05)	2.019 (−0.336, 4.798)	0.154 (−1.628, 2.129)

Bold values indicate statistical significance.

**FIGURE 7:**
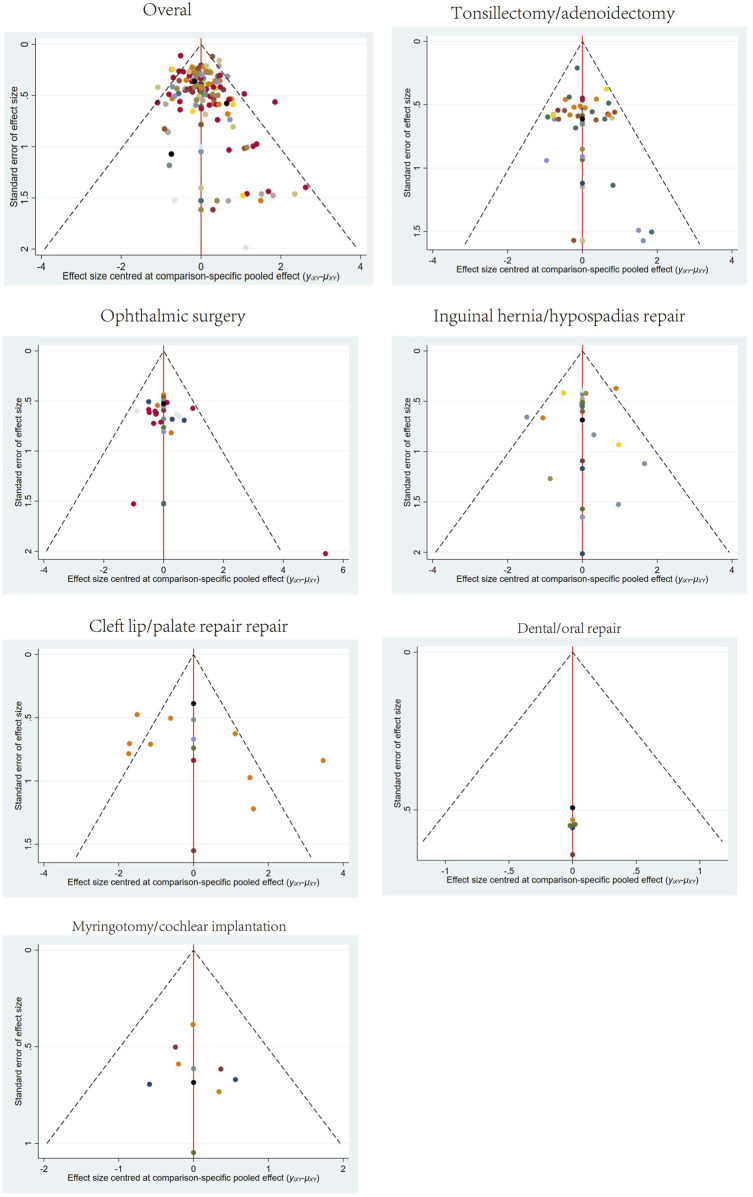
Funnel plots for different surgery sites.

Publication bias tests were conducted for different surgical sites using comparison-adjusted funnel plots. The results showed some asymmetry in the scatter points for overall surgical sites, tonsillectomy/adenoidectomy, and cleft lip/palate repair, indicating the potential presence of publication bias and small sample size effects. The funnel plots for all outcomes are presented in Figure 7.

## 4 Discussions

Sevoflurane is widely used in pediatric anesthesia due to its low blood-gas solubility, offering a smooth and predictable induction and maintenance process. However, many pediatric patients experience ED during the early recovery from sevoflurane anesthesia, a troublesome complication. Several studies have attempted to explain the mechanisms by which sevoflurane induces ED in pediatric patients. Sevoflurane mediates inhibitory postsynaptic currents by binding to gamma-aminobutyric acid receptors and exerts a biphasic effect on the central nervous system. At high concentrations, sevoflurane enhances inhibitory postsynaptic currents, producing anesthesia, but at lower concentrations, it reverses inhibitory currents, leading to agitation or delirium (Zhao et al., 2020). Additionally, a previous study demonstrated that sevoflurane directly stimulates locus coeruleus neurons, increasing the release of norepinephrine (Yasui et al., 2007). Locus coeruleus neurons are primarily involved in controlling alertness and wakefulness. Moreover, higher brain concentrations of glucose and lactate associated with sevoflurane use have been positively correlated with the incidence of ED (Jacob et al., 2012).

The incidence of ED varies between 30% and 80%, with a maximum rate reported as high as 90.5% (Wang et al., 2023). This significant variation across studies may largely be attributed to differences in the use of hypnotic agents and the surgical sites involved (Lee and Sung, 2020; Kanaya, 2016). Previous studies have shown that head and neck surgeries, including ophthalmic and ENT surgeries, are associated with a higher incidence of pediatric ED (Dahmani et al., 2010; Chen et al., 2024). Skilled anesthesiologists strive to provide a safe and comfortable anesthetic experience for patients; therefore, developing targeted anesthetic strategies tailored to specific surgical characteristics is essential. However, relevant research in this area remains limited.

Since each anesthetic adjuvant has unique benefits and potential adverse side effects (Wang et al., 2016), choosing the best single treatment can be challenging. In a previous NMA, the combination of dexmedetomidine and midazolam had the highest cumulative ranking probability and seemed to perform better than dexmedetomidine alone. Our findings are similar. We found that dexmedetomidine consistently performed well overall and across various specific surgery types, especially when combined with other drugs such as alfentanil, midazolam, and esketamine, effectively reducing the incidence of ED. Compared to previous meta-analyses that focused solely on single-drug treatments (Wang et al., 2017; Tan et al., 2019; Jiao et al., 2020), our results suggest that the efficacy of dexmedetomidine alone might have been overestimated in these studies.

Dexmedetomidine is a highly selective α-2 agonist that acts on the brain, peripheral nervous system, and spinal cord (Dahmani et al., 2010) As a highly selective α-2 agonist (Dahmani et al., 2010), dexmedetomidine possesses anxiolytic, sedative, and analgesic properties, making it the first-choice adjuvant for preventing delirium during sevoflurane anesthesia (Wang et al., 2017). The European Society of Anesthesiology guidelines recommend the use of α-2 receptor agonists, such as dexmedetomidine or clonidine, to prevent ED (Aldecoa et al., 2017). Our study shows that when dexmedetomidine is combined with alfentanil, there is a trend toward enhanced preventive effects, particularly in tonsillectomy/adenoidectomy procedures. The mechanism of alfentanil in preventing ED after sevoflurane anesthesia may be related to its analgesic and mildly sedative effects (Choi et al., 2016). One study included in this review showed that intravenous alfentanil (10 μg/kg and 20 μg/kg) administered during the induction phase of anesthesia in children undergoing adenotonsillectomy could reduce the incidence of ED (Kim et al., 2009). Another study, also in children undergoing adenotonsillectomy, used the same doses of alfentanil with an additional dose of dexmedetomidine 10 min after induction, which significantly reduced ED compared to dexmedetomidine alone (Zhang et al., 2022). These studies suggest that alfentanil may have a preventive role in ED. However, as alfentanil has a rapid onset but a short duration of action, its effect may have already faded by the time of emergence from anesthesia if administered solely during induction. A possible explanation for the observed effects in these studies could be the higher doses of alfentanil used, combined with relatively short surgery durations. In our study, however, the use of alfentanil alone did not show statistically significant results (logRR = −0.83, 95% CrI: −1.68, 0.02). Therefore, the combination effect of dexmedetomidine and alfentanil should be interpreted with caution, as the observed benefits are likely more attributable to the effects of dexmedetomidine.

This study also found that among the limited comparisons of individual or combined drugs, the combination of propofol and midazolam showed significant advantages in ophthalmic surgeries. For inguinal hernia/hypospadias repair and dental/oral repair, the available evidence suggested that the combination of dexmedetomidine and midazolam was the most effective. Fentanyl and sufentanil demonstrated high efficacy and were widely used in specific surgeries, such as ENT procedures. Drug selection should thus vary depending on the surgery type, patient condition, and needs for postoperative pain control and recovery.

Given the potential impact of different demographic characteristics, medication strategies and timing, and study quality on the results (Wang et al., 2021), we performed a network meta-regression for sensitivity analysis. The results showed that mean age (years), male percentage (%), time of prescription, and risk of bias had no significant effect on the association between anesthetic adjuvants and sevoflurane-related ED. This further supports the robustness of our main findings, which remain unaffected by these confounding factors.

Potential safety concerns or adverse effects of specific drug combinations, particularly for sedative combinations, should be addressed. In previous meta-analyses, most individual and combination treatments, including dexmedetomidine, showed no significant differences compared to the placebo group in terms of extubation time, emergence time, or duration of post anesthesia care unit stay (Wang et al., 2021; Wang et al., 2016). Additionally, in the studies included in this review regarding the combination of dexmedetomidine and alfentanil, no significant increase in respiratory depression or other adverse events was observed (Bilgen et al., 2014; Kim et al., 2009; Zhang et al., 2022).

This study has several limitations. First, literature and sample sizes for certain surgical sites, such as cleft lip/palate repair, dental/oral repair, and myringotomy/cochlear implantation, are relatively scarce, which affects the quality of the evidence. Second, although our study found that the combination of anesthetic adjuvants might be more effective in reducing ED compared to single-drug use, research on combination therapies is still relatively limited and requires further supporting evidence. Finally, clinical heterogeneity is introduced by differences in doses, administration methods, and patient age across the studies included in the literature.

## 5 Conclusion

This study comprehensively and systematically reviewed various anesthetic adjuvants and combinations to prevent sevoflurane-related ED across different surgical sites. The results indicated that overall, as well as for tonsillectomy/adenoidectomy, the combination of dexmedetomidine and alfentanil was the best option; for ophthalmic surgery, the combination of propofol and midazolam was optimal; and dexmedetomidine and midazolam showed the best effectiveness in inguinal hernia/hypospadias repair and dental/oral repair. Dexmedetomidine and fentanyl performed well in cleft lip/palate repair and myringotomy/cochlear implantation, respectively. These findings highlight the importance of selecting targeted anesthetic adjuvants based on the specific surgical site.

## Data Availability

The original contributions presented in the study are included in the article/Supplementary Material, further inquiries can be directed to the corresponding author.
