# Supplementary material for: Optimizing adjuvant strategies for sevoflurane-related emergence delirium: a Bayesian network meta-analysis in pediatric surgery
Source: Front Pharmacol. 2025 Jul 4;16:1573640. doi: 10.3389/fphar.2025.1573640 (PMC12271749; doi:10.3389/fphar.2025.1573640)
Supplement: Supplementary file 1 [file Supplementaryfile1.docx]

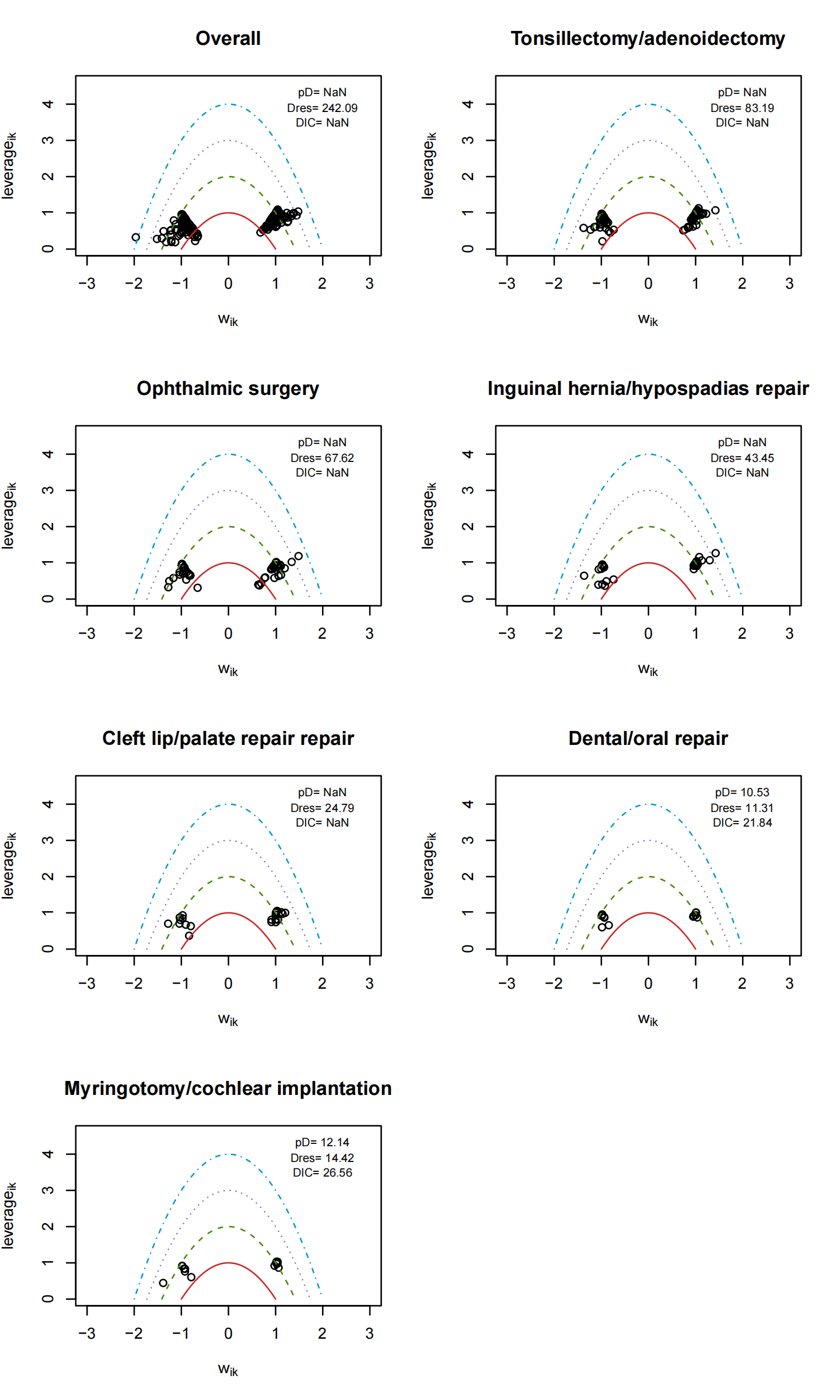


Figure S1 Leverage plots assessing model convergence

| **Table S1. Characteristics of included studies** | | | | | | | | | | | | | | | | | |
| --- | --- | --- | --- | --- | --- | --- | --- | --- | --- | --- | --- | --- | --- | --- | --- | --- | --- |
| **Study** | **Surgical site** | **Time of prescription** | **Treatment 1** | **Age (year)** | **Male (%)** | **n** | **EA cases** | **Treatment 2** | **Age (year)** | **Male (%)** | **n** | **EA cases** | **Treatment 3** | **Age (year)** | **Male (%)** | **n** | **EA cases** |
| Abbas, 2019 | Inguinal hernia repair | 3 minutes before the end of anesthesia | Placebo | 4.5 ± 1.7 | 84 | 32 | 26 | Propofol | 4.6 ± 1.4 | 91 | 32 | 5 |  |  |  |  |  |
| Abdelaziz, 2016 | Pediatric strabismus | Just before the start of surgery | Dexmedetomidine | 2.7 ± 1.5 | 52 | 35 | 4 | Midazolam | 2.5 ± 1.2 | 52 | 35 | 7 | Placebo | 2.8 ± 1.7 | 56 | 35 | 15 |
| Abdelhalim, 2013 | Adenotonsillectomy | 10 minutes before the end of surgery​ | Ketamine | 5.1 ± 1.6 | 60 | 40 | 6 | Fentanyl | 4.9 ± 1.5 | 55 | 40 | 7 | Placebo | 4.8 ± 1.8 | 60 | 40 | 17 |
| Abdelmawgoud, 2012 | Adenotonsillectomy | 1 hour before surgery​ | Ketamine | 6.6 ± 1.5 | 61 | 39 | 4 | Placebo | 6.5 ± 1.7 | 60 | 38 | 20 |  |  |  |  |  |
| Abu-Shahwan, 2007 | Dental repair | Midazolam: Before surgery Ketamine: 10 minutes before the end of anesthesia | Ketamine +midazolam | 5.3 ± 0.9 | 48 | 42 | 7 | Midazolam | 5.4 ± 0.8 | 55 | 42 | 13 |  |  |  |  |  |
| Abu-Shahwan, 2008 | Magnetic resonance imaging | Just before the end of diagnostic procedure | Propofol | 5.3 ± 0.9 | 60 | 42 | 2 | Placebo | 5.4 ± 0.8 | 56 | 42 | 11 |  |  |  |  |  |
| Akin, 2012 | Adenotonsillectomy | 45-60 minutes before the start of anesthesia and operation | Midazolam | 6.0 ± 2.0 | 60 | 45 | 5 | Dexmedetomidine | 5.0 ± 1.7 | 58 | 45 | 8 |  |  |  |  |  |
| Alansary, 2023 | Lip palate repair | During anesthesia in the operation | Dexmedetomidine | - | 64 | 25 | 4 | MgSO4 | - | 72 | 25 | 5 | Placebo | – | 76 | 25 | 7 |
| Ali, 2013 | Adenotonsillectomy | Midazolam: 30 min before separation from the parents Propofol: 5 minutes before the end of anesthesia Dexmedetomidine: 5 minutes before the end of anesthesia | Midazolam | 3.9 ± 1.6 | 55 | 40 | 22 | Propofol + midazolam | 4.2 ± 1.4 | 60 | 40 | 13 | Dexmedetomidine + midazolam | 4.3 ± 1.3 | 58 | 40 | 5 |
| [Ali, 2020](https://bmcanesthesiol.biomedcentral.com/articles/10.1186/s12871-023-02356-x" \l "ref-CR11" \o "Ali ST, Asthana V, Gupta D, Singh SK. A comparative evaluation of oral clonidine, Dexmedetomidine, and Melatonin as premedicants in Pediatric patients undergoing Subumbilical surgeries. Rom J Anaesth Intensive Care. 2020;27(1):35–42.) | Elective infraumbilical surgical procedure | During anesthesia in the operation | Melatonin | (3-8) | - | 35 | 1 | Dexmedetomidine | (3-8) | - | 35 | 0 |  |  |  |  |  |
| Almenrader, 2007 | Inguinal herniorrhaphy | Before the start of anesthesia and operation | Midazolam | 3.9 ± 1.6 | 88 | 34 | 2 | Clonidine | 3.7 ± 1.7 | 80 | 30 | 0 |  |  |  |  |  |
| Amer, 2022 | Ophthalmic surgery | 20 minutes before the end of surgery​ | Dexmedetomidine | 5.73 ± 2.592 | 60 | 40 | 2 | Propofol | 6.10 ± 2.285 | 50 | 40 | 11 |  |  |  |  |  |
| Aouad, 2007 | Strabismus surgery | Midazolam: 15–30 min before separation from the parents Propofol: Just before the end of anesthesia | Propofol + Midazolam | 4.2 ± 1.4 | 46 | 41 | 8 | Midazolam | 4.3 ± 1.3 | 58 | 36 | 17 |  |  |  |  |  |
| Asaad, 2011 | Cochlear Implantation | During anesthesia in the operation | Fentanyl | 6.8 ± 1.7 | 90 | 30 | 6 | Dexmedetomidine | 6.1 ± 1.4 | 90 | 30 | 5 | Placebo | 6.6 ± 1.3 | 93 | 30 | 12 |
| Bae, 2010 | Ophthalmic surgery | At the end of anesthesia | Midazolam | 4.9 ± 1.6 | 53 | 15.00 | 2 | Placebo | 4.1 ± 1.4 | 47 | 45.00 | 19 |  |  |  |  |  |
| Baek, 2022 | Ophthalmic surgery | During anesthesia in the operation | Remifentanil | 6.24 ± 1.09 | 56 | 45 | 15 | Fentanyl | 5.73 ± 1.37 | 56 | 45 | 12 |  |  |  |  |  |
| Bakhamees, 2009 | Adenoidectomy | Just after the induction | Placebo | 4.6 ± 1.5 | 48 | 40 | 21 | Fentanyl | 5.1 ± 1.9 | 50 | 40 | 14 |  |  |  |  |  |
| Bedirli, 2017 | Tonsillectomy/adenoidectomy | During anesthesia in the operation | Tramadol | 8.4 ± 2.1 | 69 | 39 | 3 | Dexmedetomidine | 6.7 ± 3.1 | 55 | 38 | 3 |  |  |  |  |  |
| Bergendahl, 2004 | Adeno-tonsillectomy | 30—60 min prior to induction of anaesthesia | Midazolam | 5.7 ± 1.8 | 58 | 52 | 5 | Clonidine | 5.7 ± 1.8 | 52 | 48 | 0 |  |  |  |  |  |
| Bilgen, 2014 | Urological surgery | Ketamine and alfentanil: 8-10 min before the induction of anaesthesia | Ketamine | 5.4 ± 2.2 | – | 26 | 1 | Placebo | 4.7 ± 2.7 | – | 27 | 11 | Alfentanil | 5.6 ± 2.6 | - | 25 | 9 |
| Bong, 2015 | Magnetic resonance imaging | 10 minutes after induction of anaesthesia | Dexmedetomidine | 3 (3–4) | 60 | 40 | 18 | Propofol | 4 (3–5) | 60 | 39 | 16 | Placebo | 4 (3–6 ) | 60 | 41 | 13 |
| Bromfalk, 2023 | Tonsillectomy/adenoidectomy | 60 minutes before the start of anesthesia and operation | Midazolam | 4.2 ± 0.9 | 62 | 26 | 2 | Clonidine | 4.5 ± 0.9 | 58 | 25 | 2 | Dexmedetomidine | 4.2 ± 1.0 | 57 | 30 | 0 |
| Cai, 2021 | Inguinal hernia/hypospadias repair | 30 minutes before the start of anesthesia and operation | Midazolam | 5.1 (3.0,5.8) | 92 | 37 | 10 | Dexmedetomidine | 4.3 (3.4,5.8) | 89 | 46 | 14 | Dexmedetomidine+ midazolam | 5.0 (3.4,5.7) | 89 | 46 | 7 |
| Cai, 2024 | Inguinal hernia repair | 5 minutes before the end of surgery | Remimazolam | 2.8 (1.8, 5.1) | 75 | 39 | 3 | Placebo | 2.6 (1.9, 4.5) | 19 | 40 | 14 |  |  |  |  |  |
| Chen, 2010 | Ophthalmic surgery | Just before the end of anesthesia | Midazolam + fentanyl | 3.6 ± 1.9 | - | 40 | 6 | Propofol + fentanyl | 3.6 ± 1.9 | - | 40 | 8 | Ketamine + fentanyl | 3.8 ± 2.0 | – | 40 | 18 |
| Chen, 2013 | Strabismus surgery | During anesthesia in the operation | Dexmedetomidine | 4.1 ± 1.3 | 63 | 28 | 3 | Ketamine | 4.2 ± 1.2 | 67 | 28 | 6 | Placebo | 4.3 ± 1.1 | 62 | 28 | 11 |
| Chen, 2018 | Tonsillectomy/adenoidectomy | During anesthesia in the operation | Placebo | 4 (4–5) | 85 | 20 | 6 | Dexmedetomidine | 5 (3–6) | 85 | 20 | 1 |  |  |  |  |  |
| Chen, 2023 | Tonsillectomy/adenoidectomy | Just before the end of anesthesia | Ketamine | 5.0 (1.4) | 61 | 54 | 4 | Placebo | 5.4 (1.4) | 59 | 54 | 12 |  |  |  |  |  |
| Cho, 2014 | Strabismus | Just before the end of surgery | Midazolam | 8.0 ± 2.1 | 33 | 60 | 10 | Placebo | 8.0 ± 2.1 | 30 | 30 | 13 |  |  |  |  |  |
| Choi, 2016 | Ophthalmic surgery | Remifentanil: During anesthesia in the operation Alfentanil: 10 min before the end of the surgery | Placebo | 6.1 ± 2.3 | 48 | 33 | 21 | Remifentanil | 6.2 ± 2.0 | 50 | 34 | 11 | Remifentanil + alfentanil | 5.8 ± 1.9 | 46 | 35 | 11 |
| Costi, 2015 | Magnetic resonance imaging | During anesthesia in the operation | Propofol + midazolam | 4.0 ± 2.4 | 53 | 109 | 8 | Midazolam | 4.1 ± 2.8 | 56 | 109 | 32 |  |  |  |  |  |
| Cravero, 2003 | Magnetic resonance imaging | Just before the end of anesthesia | Fentanyl | 4.8 ± 3.0 | 50 | 16 | 2 | Placebo | 4.3 ± 2.5 | 50 | 16 | 9 |  |  |  |  |  |
| Dalens, 2006 | Magnetic resonance imaging | Just before the end of anesthesia | Ketamine | 3.0 ± 1.6 | – | 33 | 0 | Placebo | 2.61 ± 1.7 | – | 28 | 3 |  |  |  |  |  |
| Demirbilek, 2004 | Tonsillectomy/adenoidectomy | Midazolam: Before surgery Fentanyl: During anesthesia in the operation | Fentanyl + midazolam | 5.0 ± 1.4 | 50 | 30 | 2 | Midazolam | 5.3 ± 1.5 | 63 | 30 | 4 |  |  |  |  |  |
| Di, 2014 | Lip/cleft palate repair | 30 min before the end of surgery | Dexmedetomidine | (0.5-3) | - | 30 | 4 | Placebo | (0.5-3) | - | 17 | 4 |  |  |  |  |  |
| Di, 2017 | Tonsillectomy/adenoidectomy | 10 minutes before the start of anesthesia | Placebo | 5.3 ± 1.2 | 56 | 20 | 6 | Dexmedetomidine | 4.7 ± 1.0 | 40 | 25 | 0 |  |  |  |  |  |
| Dong, 2010 | Adenotonsillectomy | During anesthesia in the operation | Placebo | 5.9 ± 2.1 | 43 | 30 | 20 | Remifentanil | 5.9 ± 1.8 | 47 | 30 | 7 |  |  |  |  |  |
| Erdil, 2009 | Adenoidectomy | During anesthesia in the operation | Fentanyl | 4.6 ± 1.4 | 33 | 30 | 7 | Dexmedetomidine | 4.7 ± 1.4 | 57 | 30 | 9 | Placebo | 4.2 ± 1.3 | 53 | 30 | 20 |
| Finkel, 2001 | Bilateral myringotomy | During anesthesia in the operation | Fentanyl | 2.1 ± 1.4 | 60 | 101 | 19 | Placebo | 2.5 ± 1.5 | 65 | 49 | 21 |  |  |  |  |  |
| Galinkin, 2000 | Bilateral myringotomy | Just before the start of surgery | Fentanyl | 2.0 ± 1.2 | 66 | 64 | 1 | Midazolam | 2.0 ± 1.2 | 71 | 69 | 16 |  |  |  |  |  |
| Ghai, 2010 | Ophthalmic surgery | Just after the induction | Clonidine | 3.4 ± 1.5 | 62 | 39 | 3 | Placebo | 3.0 ± 1.4 | 60 | 40.00 | 16 |  |  |  |  |  |
| Ghosh, 2011 | Urogenital and lower limb surgery | During anesthesia in the operation | Clonidine | 3.7 ± 1 | 73 | 60 | 10 | Placebo | 3.7 ± 1 | 70 | 30 | 12 |  |  |  |  |  |
| Golmohammadi, 2024 | Tonsillectomy/adenoidectomy | 10 minutes after induction of anaesthesia | Dexmedetomidine | 3.97 ± 1.04 | 68 | 38 | 6 | Placebo | 3.62 ± 1.12 | 52 | 38 | 8 |  |  |  |  |  |
| Guler, 2005 | Adenotonsillectomy | 5 min before the end of anesthesia | Dexmedetomidine | 4.7 ± 1.2 | 63 | 30 | 5 | Placebo | 4.5 ± 1.2 | 70 | 30 | 17 |  |  |  |  |  |
| Hadi, 2015 | Adenotonsillectomy | 10 minutes before the end of anesthesia | Dexmedetomidine + ketamine | 4.2 ± 1.3 | 51 | 45 | 5 | Placebo | 4.2 ± 1.1 | 51 | 47 | 22 |  |  |  |  |  |
| Hauber, 2015 | Tonsillectomy/adenoidectomy | 5 minutes before the end of surgery | Dexmedetomidine | 6.1 ± 1.6 | 51 | 195 | 69 | Placebo | 5.8 ± 1.6 | 51 | 198 | 125 |  |  |  |  |  |
| He, 2023 | Dental repair | 30 minutes before the induction of anesthesia | Placebo | 4.8 ± 0.75 | 67 | 30 | 21 | Dexmedetomidine | 4.8±0.85 | 50 | 30 | 11 |  |  |  |  |  |
| Heinmiller, 2013 | Ophthalmic surgery | Just before the start of surgery | Clonidine | 4.3 ± 1.5 | 40 | 25.00 | 6 | Placebo | 4.1 ± 1.3 | 48 | 25.00 | 15 |  |  |  |  |  |
| Huang, 2022 | Cleft palate repair | 30 minutes before the end of surgery | Placebo | - | - | 60 | 24 | Propofol | - | - | 61 | 17 | Dexmedetomidine | – | – | 61 | 6 |
| Ibacache, 2004 | Inguinal hernia repair | During anesthesia in the operation | Dexmedetomidine | 3.8 ± 1.5 | 87 | 60 | 8 | Placebo | 4.4 ± 1.9 | 97 | 30 | 4 |  |  |  |  |  |
| Ibrahim, 2023 | Tonsillectomy/adenoidectomy | Just before the end of anesthesia | Ketamine | - | - | 30 | 0 | Nalbuphine | - | - | 30 | 2 | Placebo | – | – | 30 | 2 |
| Isik, 2006 | Magnetic resonance imaging | Just after the induction | Dexmedetomidine | 4.4 ± 1.2 | 76 | 21 | 1 | Placebo | 4.1 ± 2.6 | 57 | 21 | 10 |  |  |  |  |  |
| Jangra, 2022 | Ophthalmic surgery | 10 minutes before the end of surgery | Melatonin | 5.5 ± 2.2 | 62 | 60 | 22 | Dexmedetomidine | 5.4 ± 2.0 | 63 | 60 | 10 |  |  |  |  |  |
| Jayaraj, 2023 | Tonsillectomy/adenoidectomy | 10 minutes before the end of surgery | Ketamine + propofol | - | 53 | 45 | 12 | Dexmedetomidine | - | 49 | 45 | 9 |  |  |  |  |  |
| Jeong, 2012 | Ophthalmic surgery | 10 min before the end of the surgery | Placebo | 4.8 ± 0.4 | 55 | 20.00 | 15 | Ketamine | 5.0 ± 0.4 | 50 | 40.00 | 10 |  |  |  |  |  |
| Ju, 2013 | Lip/cleft palate repair | During anesthesia in the operation | Dexmedetomidine | (0.5-3) | - | 40 | 3 | Placebo | (0.5-3) | - | 40 | 9 |  |  |  |  |  |
| Jun, 2018 | Lip/cleft palate repair | During anesthesia in the operation | Dexmedetomidine | 1.71±0.61 | - | 110 | 7 | Placebo | 1.74±0.62 | - | 110 | 16 |  |  |  |  |  |
| Jung, 2010 | Ophthalmic surgery | Just after the induction | Ketamine | 5.4 ± 1.9 | 48 | 23 | 4 | Fentanyl | 7.5 ± 2.0 | 33 | 24 | 0 |  |  |  |  |  |
| Kawai, 2019 | Dental repair | 30 minutes before the end of surgery | Midazolam | 6.1±2.4 | 68 | 40 | 10 | Placebo | 5.9±1.8 | 75 | 40 | 14 |  |  |  |  |  |
| Khalifa, 2013 | Adenotonsillectomy | During anesthesia in the operation | Placebo | 5.6 ± 1.2 | 53 | 30 | 18 | Ketamine | 5.9 ± 1.6 | 53 | 30 | 6 |  |  |  |  |  |
| Kim, 2009 | Adenotonsillectomy | 1 min after loss of the eyelash reflex | Placebo | 6.5 (3–10) | 88 | 30 | 24 | Alfentanil | 7 (3–10) | 53 | 66 | 23 |  |  |  |  |  |
| Kim, 2011 | Ophthalmic surgery | 5 minutes before the end of anesthesia | Midazolam | 6.0 ± 2.2 | 43 | 35 | 15 | Propofol | 6.3 ± 2.6 | 42 | 31 | 15 | Placebo | 6.7 ± 1.2 | 60 | 35 | 26 |
| Kim, 2013 | Inguinal hernia repair | 10 minutes before the end of surgery | Propofol | 3.6 (1.8–6.0) | 75 | 69 | 16 | Fentanyl | 3.7 (1.5–6.0) | 58 | 66 | 13 | Placebo | 3.8 (1.7–5.9) | 69 | 70 | 35 |
| Kim, 2014 | Ambulatory hernioplasty | During anesthesia in the operation | Dexmedetomidine | 1.4 ± 0.5 | – | 20 | 1 | Placebo | 1.4 ± 0.9 | – | 20 | 11 |  |  |  |  |  |
| Kim, 2016 | Ophthalmic surgery | One hour before the start of anesthesia and operation in the waiting area | Midazolam | 4.1 ± 1.4 | 47 | 34 | 15 | Ketamine | 4.2 ± 1.3 | 48 | 33 | 11 |  |  |  |  |  |
| Komazaki, 2020 | Tonsillectomy | Before the start of anesthesia and operation | Melatonin | (1.5-10) | - | 24 | 16 | Placebo | (1.5-10) | - | 24 | 16 |  |  |  |  |  |
| Lankinen, 2006 | Adenoidectomy | During anesthesia in the operation | Clonidine | 2.6 ± 1.7 | – | 24 | 13 | Placebo | 3.0 ± 1.8 | – | 26 | 16 |  |  |  |  |  |
| Lee, 2010 (1) | Tonsillectomy/adenoidectomy | 10 minutes before the end of surgery | Ketamine | 6.1 ± 0.5 | 57 | 60 | 12 | Placebo | 7.0 ± 0.7 | 67 | 30 | 24 |  |  |  |  |  |
| Lee, 2010 (2) | Adenotonsillectomy | At the end of anesthesia | Propofol | 5.8 ± 1.9 | 59 | 44 | 27 | Placebo | 6.3 ± 1.6 | 57 | 44 | 30 |  |  |  |  |  |
| Li, 2011 | Adenotonsillectomy | During anesthesia in the operation | Placebo | 6.4 ± 1.8 | 70 | 34 | 24 | Sufentanil | 6.4 ± 1.8 | 65 | 34 | 15 | Fentanyl | 6.4 ± 1.8 | 66 | 32 | 4 |
| Li, 2013 | Elective repair | Just before the start of surgery | Fentanyl | 3.5 ± 1.2 | 82 | 40 | 20 | Sufentanil | 3.4 ± 1.3 | 80 | 40 | 11 |  |  |  |  |  |
| Li, 2024 | Cochlear Implantation | 15–30 minutes before the end of surgery | Nalbuphine | 1.9 ± 0.83 | 46 | 26 | 8 | Placebo | 1.9 ±0.71 | 69 | 26 | 19 |  |  |  |  |  |
| Liang, 2014 | Ophthalmic surgery | 20 min before the end of the surgery | Placebo | 5.5 ± 1.4 | 43 | 30 | 19 | Sufentanil | 5.1 ± 1.3 | 57 | 30 | 9 | Fentanyl | 4.7 ± 1.3 | 46 | 30 | 11 |
| Lili, 2012 | Ophthalmic surgery | During anesthesia in the operation | Dexmedetomidine | 5 ± 2 | 53 | 30 | 3 | Placebo | 4 ± 1 | 57 | 30 | 13 |  |  |  |  |  |
| Lin, 2016 | Cataract surgery | 45 minutes before the start of anesthesia | Dexmedetomidine | 4.7 ± 1.9 | 60 | 60 | 10 | Placebo | 4.1 ± 1.6 | 50 | 30 | 24 |  |  |  |  |  |
| Lin, 2017 | Oral operation | During anesthesia in the operation | Dexmedetomidine | 4 | 52 | 40 | 6 | Placebo | 4 | 48 | 40 | 17 |  |  |  |  |  |
| Liu, 2022 | Ophthalmic surgery | 10 minutes before surgery​ | Ketamine | 4 (3,6) | - | 30 | 4 | Placebo | 5 (3, 6) | - | 30 | 18 |  |  |  |  |  |
| Lundblad, 2015 | Inguinal hernia repair | During anesthesia in the operation | Dexmedetomidine | 5.3 ± 1.2 | 77 | 22 | 0 | Placebo | 5.5 ± 1.1 | 62 | 21 | 4 |  |  |  |  |  |
| Luo, 2017 | Lip/cleft palate repair | 30 min before the end of surgery | Dexmedetomidine | (1-5) | - | 46 | 4 | Placebo | (1-5) | - | 47 | 36 |  |  |  |  |  |
| Meng, 2012 | Tonsillectomy | Midazolam: Before surgery Dexmedetomidine: During anesthesia in the operation | Dexmedetomidine+ midazolam | 7.0 ± 2.0 | 52 | 80 | 8 | Midazolam | 7.0 ± 2.0 | 55 | 40 | 8 |  |  |  |  |  |
| Mizrak, 2010 | Ophthalmic surgery | During anesthesia in the operation | Ketamine | 7.7 ± 3.1 | 37 | 30.00 | 5 | Propofol | 6.9 ± 3.0 | 40 | 30.00 | 5 |  |  |  |  |  |
| Mizrak, 2011 | Ophthalmic surgery | Before surgery​ | Dexmedetomidine | 8.5 ± 2.6 | 50 | 30.00 | 6 | Placebo | 8.6 ± 2.8 | 43 | 30.00 | 16 |  |  |  |  |  |
| Moawad, 2015 | Magnetic resonance imaging | During anesthesia in the operation | Placebo | 4.6 ± 1.4 | 60 | 40 | 7 | Ketamine | 4.3 ± 1.4 | 55 | 80 | 7 |  |  |  |  |  |
| Mohamed, 2024 | Inguinal hernia/hypospadias repair | 10 minutes before the end of surgery | Nalbuphine | 4.26 ± 0.90 | 62 | 45 | 7 | Dexmedetomidine | 4.27 ± 0.89 | 58 | 45 | 6 | Placebo | 4.14 ± 0.84 | 60 | 45 | 18 |
| Na, 2013 | Adenotonsillectomy | During anesthesia in the operation | Remifentanil | 5.1 ± 1.3 | 64 | 42 | 15 | Placebo | 5.0 ± 1.2 | 71 | 42 | 27 |  |  |  |  |  |
| Patel, 2010 | Tonsillectomy/adenoidectomy | During anesthesia in the operation | Fentanyl | 3.8 ± 1.5 | 57 | 61 | 36 | Dexmedetomidine | 4.2 ± 2.1 | 57 | 61 | 20 |  |  |  |  |  |
| Peng, 2015 | Cleft palate repair | During anesthesia in the operation | Dexmedetomidine | 12.5 ± 3.8 | 65 | 20 | 3 | Placebo | 11.3 ± 4.5 | 70 | 20 | 18 |  |  |  |  |  |
| Pestieau, 2011 | Myringotomy | During anesthesia in the operation | Dexmedetomidine | 1.7 ± 0.9 | 70 | 51 | 14 | Fentanyl | 1.8 ± 1.2 | 56 | 23 | 3 | Placebo | 1.6 ± 1.5 | 63 | 27 | 11 |
| Rashad, 2014 | Hypospadias repair | Before the end of anesthesia and operation | Propofol | 2.3 ± 0.8 | – | 20 | 1 | Ketamine | 2.3 ± 0.8 | – | 20 | 4 | Fentanyl | 2.3 ± 0.8 | – | 20 | 2 |
| Saadawy, 2009 | Unilateral inguinal hernia repair | During anesthesia in the operation | Dexmedetomidine | 2.4 ± 0.6 | – | 30 | 2 | Placebo | 2.8 ± 1.6 | – | 30 | 9 |  |  |  |  |  |
| Sahmeddini, 2024 | Ophthalmic surgery | Before the end of anesthesia and operation | Dexmedetomidine | 5.15 ± 1.5 | 40 | 20 | 0 | Placebo | 5.32 ± 1.28 | 30 | 20 | 20 | Remifentanil | 5.21 ± 1.55 | 50 | 20 | 16 |
| Shen, 2012 | Cochlear implantation | During anesthesia in the operation | Placebo | 3.1 ± 1.4 | 56 | 25 | 6 | Remifentanil | 3.3 ± 1.3 | 52 | 25 | 5 |  |  |  |  |  |
| Sheta, 2014 | Complete dental rehabilitation | 45-60 minutes before the start of anesthesia and operation | Dexmedetomidine | 3.9 ± 0.9 | 42 | 36 | 4 | Midazolam | 4.2 ± 1.0 | 44 | 36 | 11 |  |  |  |  |  |
| Shi, 2019 | Tonsillectomy/adenoidectomy | During anesthesia in the operation | Dexmedetomidine | 5.0(4.0-6.0) | 73 | 45 | 14 | Placebo | 5.0(4.0-6.0) | 75 | 45 | 24 |  |  |  |  |  |
| Soliman, 2015 | Adenotonsillectomy | During anesthesia in the operation | Dexmedetomidine | 8.6 ± 3.1 | 48 | 75 | 6 | Placebo | 8.4 ± 3.0 | 53 | 75 | 29 |  |  |  |  |  |
| Song, 2016 | Ophthalmic surgery | Just after the induction | Dexmedetomidine | 4.3 ± 1.7 | 50 | 28.00 | 6 | Placebo | 3.8 ± 1.5 | 50 | 28.00 | 17 |  |  |  |  |  |
| Sousa-Júnior, 2021 | Tonsillectomy/adenoidectomy | During anesthesia in the operation | Clonidine | 5.44 ± 2.25 | - | 29 | 5 | Placebo | 6.0 ± 2.7 | - | 33 | 19 |  |  |  |  |  |
| Thomas, 2015 | Herniotomy | Just after the induction | Ketamine | 4.8 ± 2.3 | 89 | 38 | 12 | Fentanyl | 4.5 ± 2.6 | 84 | 76 | 36 |  |  |  |  |  |
| Vettuvanthodi, 2024 | Urogenital surgery | Just before the end of surgery | Dexmedetomidine | 4.19±0.78 | 87 | 70 | 10 | Propofol | 4.03±0.71 | 87 | 70 | 22 |  |  |  |  |  |
| Viitanen, 1999 | Ambulatory adenoidectomy | 30 minutes before the start of anesthesia and operation | Midazolam | 1.4 ± 0.7 | – | 30 | 18 | Placebo | 1.5 ± 0.6 | – | 30 | 14 |  |  |  |  |  |
| Xi, 2012 | Lip/cleft palate repair | During anesthesia in the operation | Dexmedetomidine | (1–3) | - | 15 | 1 | Placebo | (1–3) | - | 15 | 12 |  |  |  |  |  |
| Xiao, 2012 | Lip/cleft palate repair | During anesthesia in the operation | Fentanyl | 1.22±0.22 | 56 | 18 | 3 | Ketamine | 1.26±0.24 | 50 | 18 | 3 | Dexmedetomidine | 1.25±0.23 | 61 | 18 | 0 |
| Xing, 2024 | Dental repair | During anesthesia in the operation | Dexmedetomidine | 3.9 ± 1.3 | 47 | 38 | 15 | dexmedetomidine+esketamine | 3.7 ± 1.1 | 42 | 36 | 6 |  |  |  |  |  |
| Yang, 2022 | Tonsillectomy/adenoidectomy | Before the end of anesthesia and operation | Remimazolam | 5 [4–6] | 59 | 51 | 6 | Placebo | 5 [5–6] | 64 | 50 | 22 |  |  |  |  |  |
| Yao, 2015 | Ophthalmic surgery | 45 minutes before the start of anesthesia | Placebo | 4.7 ± 0.9 | 60 | 29 | 14 | Dexmedetomidine | 4.5 ± 0.8 | 63 | 30 | 5 |  |  |  |  |  |
| Yun, 2016 | Lip/cleft palate repair | During anesthesia in the operation | Dexmedetomidine | (0.5-3) | - | 60 | 3 | Placebo | (0.5-3) | - | 60 | 57 |  |  |  |  |  |
| Zhang, 2022 | Tonsillectomy/adenoidectomy | Dexmedetomidine:10 minutes after induction of anaesthesia Alfentanil: at the induction of anesthesia | Placebo | 4.53 ± 1.32 | 50 | 20 | 10 | Dexmedetomidine | 4.81 ± 1.09 | 60 | 20 | 5 | Dexmedetomidine + alfentanil | 5.13 ± 1.29 | 55 | 20 | 1 |

| Table S2. Quality assessment of risk of bias for the included studies | | | | | | | | |
| --- | --- | --- | --- | --- | --- | --- | --- | --- |
| Study | Random sequence generation | Allocation concealment | Blinding of participants and personnel | Blinding of outcome assessment | Incomplete outcome data | Selective reporting | Other bias | Overall |
| Abbas, 2019 | Low | Low | High | Low | Low | Low | High | High |
| Abdelaziz, 2016 | Low | Low | Low | Low | Low | Low | Low | Low |
| Abdelhalim, 2013 | Low | Low | Low | Low | Low | Low | Low | Low |
| Abdelmawgoud, 2012 | Low | Low | Low | Low | Low | Low | Low | Low |
| Abu-Shahwan, 2007 | Low | Low | Low | Low | Low | Low | Low | Low |
| Abu-Shahwan, 2008 | Low | Low | Low | Low | Low | Low | Low | Low |
| Akin, 2012 | High | Low | Low | Low | Low | Low | Low | High |
| Alansary, 2023 | Low | Low | Low | Some concerns | Low | Low | Low | Some concerns |
| Ali, 2013 | High | Low | Low | Low | High | Low | High | High |
| Ali, 2020 | Low | Low | Low | Some concerns | Low | Low | Low | Some concerns |
| Almenrader, 2007 | High | Low | Low | Some concerns | Low | Low | Low | High |
| Amer, 2022 | Low | Low | Low | Some concerns | Low | Low | Low | Some concerns |
| Aouad, 2007 | Low | Low | Low | Low | Low | Low | Low | Low |
| Assad, 2011 | Low | Low | Low | Low | Low | Low | High | High |
| Bae, 2010 | Low | Low | Low | Some concerns | Low | Low | Low | Some concerns |
| Baek, 2022 | Low | Low | Low | Some concerns | Low | Low | Low | Some concerns |
| Bakbamees, 2009 | High | Some concerns | Low | Some concerns | Low | Low | Low | High |
| Bedirli, 2017 | Low | Low | Low | Some concerns | Low | Low | Low | Some concerns |
| Bergendahl, 2004 | Low | Low | Low | Low | Low | Low | Low | Low |
| Bilgen, 2014 | Low | Low | Low | Low | Low | Low | Low | Low |
| Bong, 2015 | Low | Low | Low | Some concerns | Low | Low | Low | Some concerns |
| Bromfalk, 2023 | Low | Low | Low | Some concerns | Low | Low | Low | Some concerns |
| Cai, 2021 | Low | Low | Low | Some concerns | Low | Low | Low | Some concerns |
| Cai, 2024 | Low | Low | Low | Some concerns | Low | Low | Low | Some concerns |
| Chen, 2010 | Low | Low | Low | Some concerns | Low | Low | Low | Some concerns |
| Chen, 2013 | Low | Some concerns | Low | Some concerns | Low | High | Low | High |
| Chen, 2018 | Low | High | Low | Some concerns | Low | Low | Low | High |
| Chen, 2023 | Low | Low | Low | Some concerns | Low | Low | Low | Some concerns |
| Cho, 2014 | Low | Low | Low | Low | Low | Low | Low | Low |
| Choi, 2016 | Low | Low | Low | Low | Low | Low | Low | Low |
| Costi, 2015 | Low | Low | Low | Low | Low | Low | Low | Low |
| Cravero, 2003 | Low | Low | Low | Low | Low | Low | High | High |
| Dalens, 2006 | Low | Low | Low | Low | Low | Low | Low | Low |
| Demirbilek, 2004 | Low | Low | Low | Some concerns | Low | Low | Low | Some concerns |
| Di, 2014 | Low | Low | Low | Some concerns | Low | Low | Low | Some concerns |
| Di, 2017 | Low | Low | Low | Some concerns | Low | Low | Low | Some concerns |
| Dong, 2010 | Low | Low | Low | Low | Low | Low | High | High |
| Erdil, 2009 | Low | Low | Low | Some concerns | Low | Low | Low | Some concerns |
| Finkel, 2001 | Low | Low | Low | Low | Low | Low | Low | Low |
| Galinkin, 2000 | Low | Low | Low | Low | Low | Low | Low | Low |
| Ghai, 2010 | Low | Low | Low | Some concerns | Low | Low | Low | Some concerns |
| Ghosh, 2011 | Low | Low | Low | Low | Low | Low | Low | Low |
| Golmohammadi, 2024 | Low | Low | Low | Some concerns | Low | Low | Low | Some concerns |
| Guler, 2005 | Low | Low | Low | Low | Low | Low | Low | Low |
| Hadi, 2015 | High | Low | Low | Some concerns | Low | Low | Some concerns | High |
| Hauber, 2015 | Low | Low | Low | Some concerns | Low | Low | Low | Some concerns |
| He, 2023 | Low | Low | Low | Some concerns | Low | Low | Low | Some concerns |
| Heinmiller, 2013 | Low | Low | Low | Some concerns | Low | Low | Low | Some concerns |
| Huang, 2022 | Low | Low | Low | Some concerns | Low | Low | Low | Some concerns |
| Ibacache, 2004 | Low | Some concerns | Low | Some concerns | Low | Low | Some concerns | Some concerns |
| Ibrahim, 2023 | Low | Low | Low | Some concerns | Low | Low | Low | Some concerns |
| Isik, 2006 | Low | Some concerns | Low | Low | High | Low | High | High |
| Jangra, 2022 | Low | Low | Low | Some concerns | Low | Low | Low | Some concerns |
| Jayaraj, 2023 | Low | Low | Low | Some concerns | Low | Low | Low | Some concerns |
| Jeong, 2012 | Low | Low | Low | Some concerns | Low | Low | Low | Some concerns |
| Ju, 2013 | Low | Low | Low | Some concerns | Low | Low | Low | Some concerns |
| Jun, 2018 | Low | Low | Low | Some concerns | Low | Low | Low | Some concerns |
| Jung, 2010 | Low | Low | Low | Low | Low | Low | Low | Low |
| Kawai, 2019 | Low | Low | Low | Some concerns | Low | Low | Low | Some concerns |
| Khalifa, 2013 | Low | Low | Low | Low | Low | Low | Low | Low |
| Kim, 2009 | Low | Low | Low | Low | Low | Low | Low | Low |
| Kim, 2011 | Low | Low | Low | Low | Low | Low | Low | Low |
| Kim, 2013 | Low | Low | Low | Some concerns | Low | Low | Low | Some concerns |
| Kim, 2014 | Low | Low | Low | Low | Low | Low | Low | Low |
| Kim, 2016 | Low | Low | Low | Low | Low | Low | Low | Low |
| Komazaki, 2020 | Low | Low | Low | Some concerns | Low | Low | Low | Some concerns |
| Lankinen, 2006 | Low | Low | Low | Low | Low | Low | Low | Low |
| Lee, 2010 (1) | Low | Low | Low | Some concerns | Low | Low | Low | Some concerns |
| Lee, 2010 (2) | Low | Low | Low | Some concerns | High | Low | Low | High |
| Li, 2011 | Low | Low | Low | Some concerns | High | Low | High | High |
| Li, 2013 | Low | Low | Low | Low | Low | Low | Low | Low |
| Li, 2024 | Low | Low | Low | Some concerns | Low | Low | Low | Some concerns |
| Liang, 2014 | Low | Low | Low | Low | Low | Low | Low | Low |
| Lili, 2012 | Low | Low | Low | Some concerns | High | Low | High | High |
| Lin, 2016 | Low | Low | High | Low | Low | Low | Low | High |
| Lin, 2017 | Low | Low | Low | Some concerns | High | High | Some concerns | High |
| Liu, 2022 | Low | Low | Low | Some concerns | Low | Low | Low | Some concerns |
| Lundblad, 2015 | Low | Low | Low | Low | Low | Low | Low | Low |
| Luo, 2017 | Low | Low | Low | Some concerns | Low | Low | Low | Some concerns |
| Meng, 2012 | Low | Low | Low | Low | Low | Low | Some concerns | Some concerns |
| Mizrak, 2010 | Low | Low | Low | Some concerns | Low | Low | Low | Some concerns |
| Mizrak, 2011 | Low | Low | Low | Some concerns | Low | Low | Low | Some concerns |
| Moawad, 2015 | Low | Low | Low | Some concerns | Low | Low | Low | Some concerns |
| Mohamed, 2024 | Low | Low | Low | Some concerns | Low | Low | Low | Some concerns |
| Na, 2013 | Low | Low | Low | Low | Low | Low | Low | Low |
| Patel, 2010 | Low | Low | Low | Some concerns | Low | Low | Low | Some concerns |
| Peng, 2015 | High | Low | Low | Some concerns | Low | Low | Low | High |
| Pestieau, 2011 | Low | Low | Low | Low | Low | Low | Low | Low |
| Rashad, 2014 | Low | Low | Low | Some concerns | Low | Low | Low | Some concerns |
| Saadawy, 2009 | Low | Low | Low | Low | Low | Low | Low | Low |
| Sahmeddini, 2024 | Low | Low | Low | Some concerns | Low | Low | Low | Some concerns |
| Shen, 2012 | Low | Low | Low | Some concerns | High | Low | Low | High |
| Sheta, 2014 | Low | Low | Low | Low | Low | Low | Low | Low |
| Shi, 2019 | Low | Low | Low | Low | Low | High | Low | High |
| Soliman, 2015 | Low | Low | Low | Low | Low | Low | Low | Low |
| Song, 2016 | Low | Low | Low | Some concerns | Low | Low | Low | Some concerns |
| Sousa-Júnior, 2021 | Low | Low | Low | Some concerns | Low | Low | Low | Some concerns |
| Thomas, 2015 | Low | Low | Low | Low | Low | Low | Low | Low |
| Vettuvanthodi, 2024 | Low | Low | Low | Some concerns | Low | Low | Low | Some concerns |
| Viitanen, 1999 | Low | Low | Low | Low | Low | Some concerns | Low | Some concerns |
| Xi, 2012 | Low | Low | Low | Some concerns | Low | Low | Low | Some concerns |
| Xiao, 2012 | Low | Low | Low | Some concerns | Low | Low | Low | Some concerns |
| Xing, 2024 | Low | Low | Low | Some concerns | Low | Low | Low | Some concerns |
| Yang, 2022 | Low | Low | Low | Some concerns | Low | Low | Low | Some concerns |
| Yao, 2015 | Low | Low | Low | Some concerns | Low | Low | Low | Some concerns |
| Yun, 2016 | Low | Low | Low | Some concerns | Low | Low | Low | Some concerns |
| Zhang, 2022 | Low | Low | Low | Some concerns | Low | Low | Low | Some concerns |


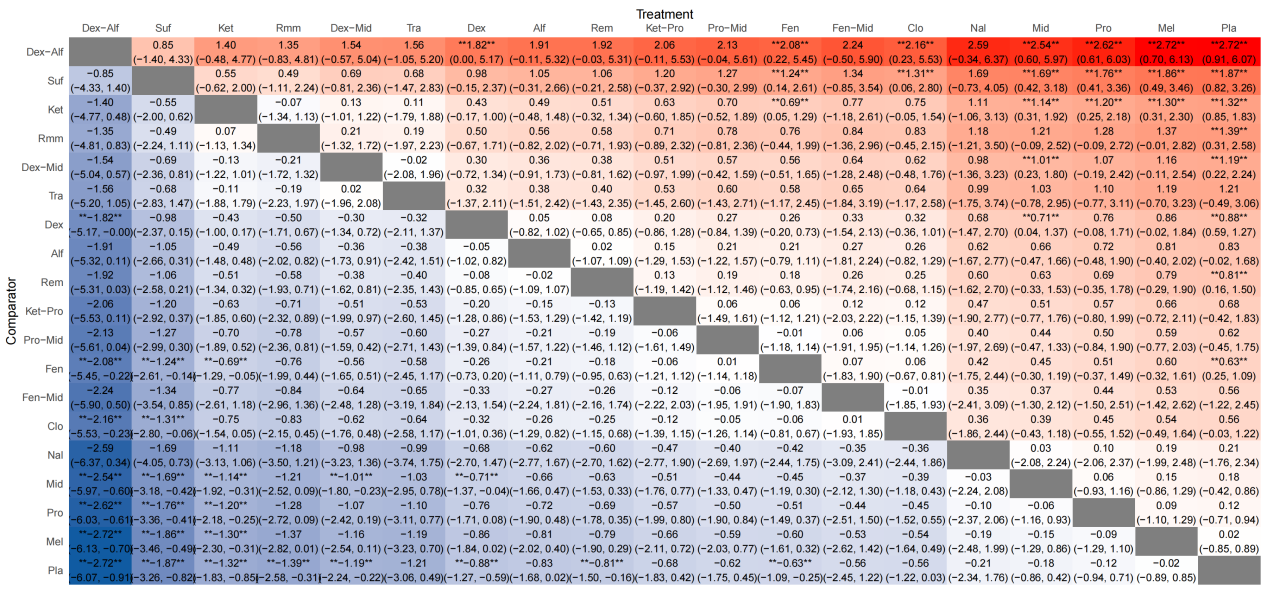


**Figure S2. League plots for tonsillectomy/adenoidectomy**


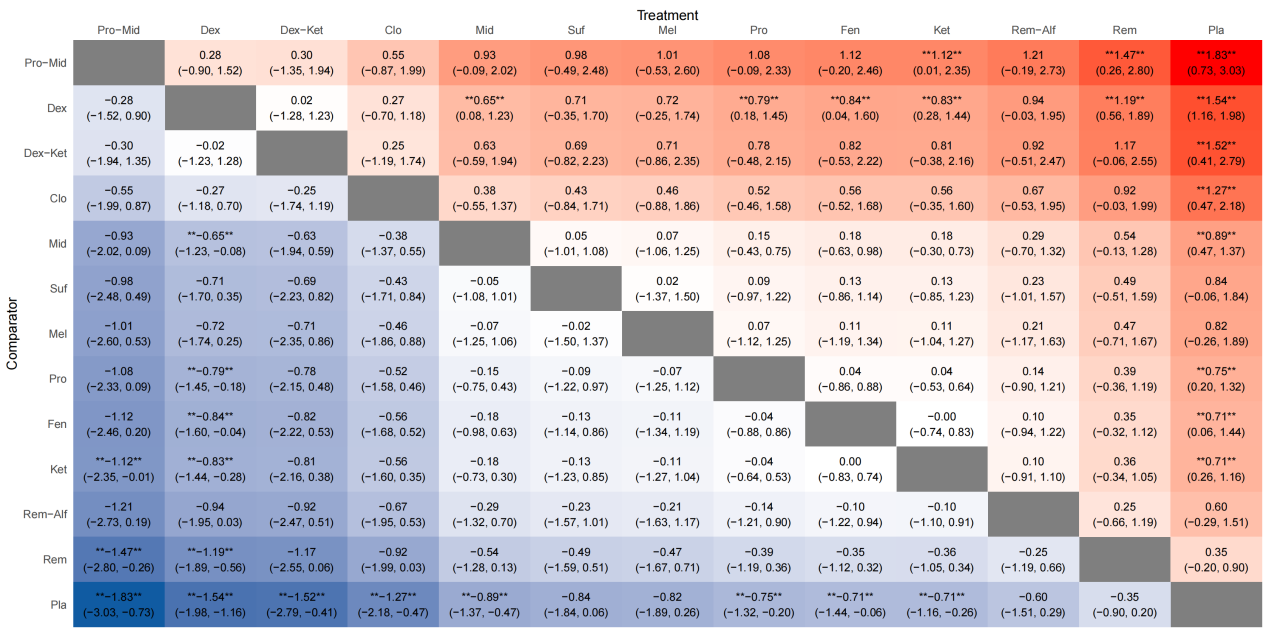


**Figure S3. League plots for ophthalmic surgery**


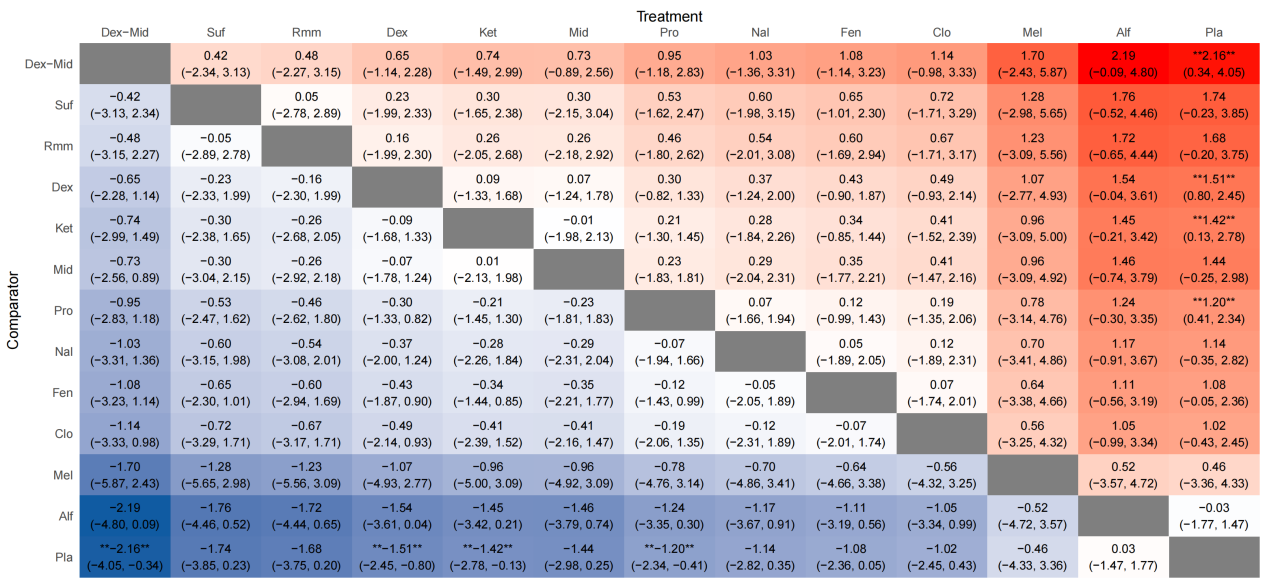


**Figure S4. League plots for inguinal hernia/hypospadias repair**


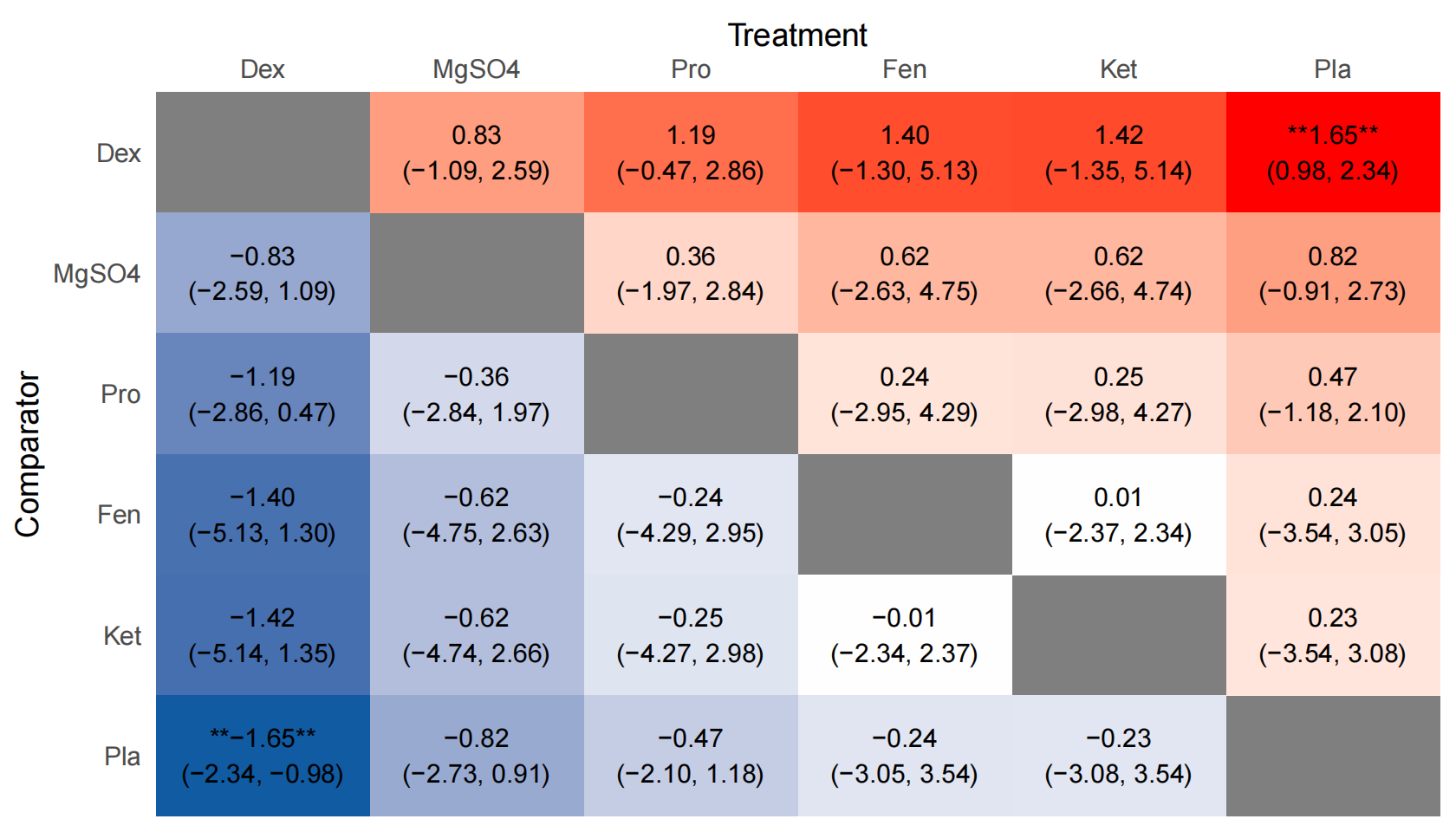


**Figure S5. League plots for cleft lip/palate repair**


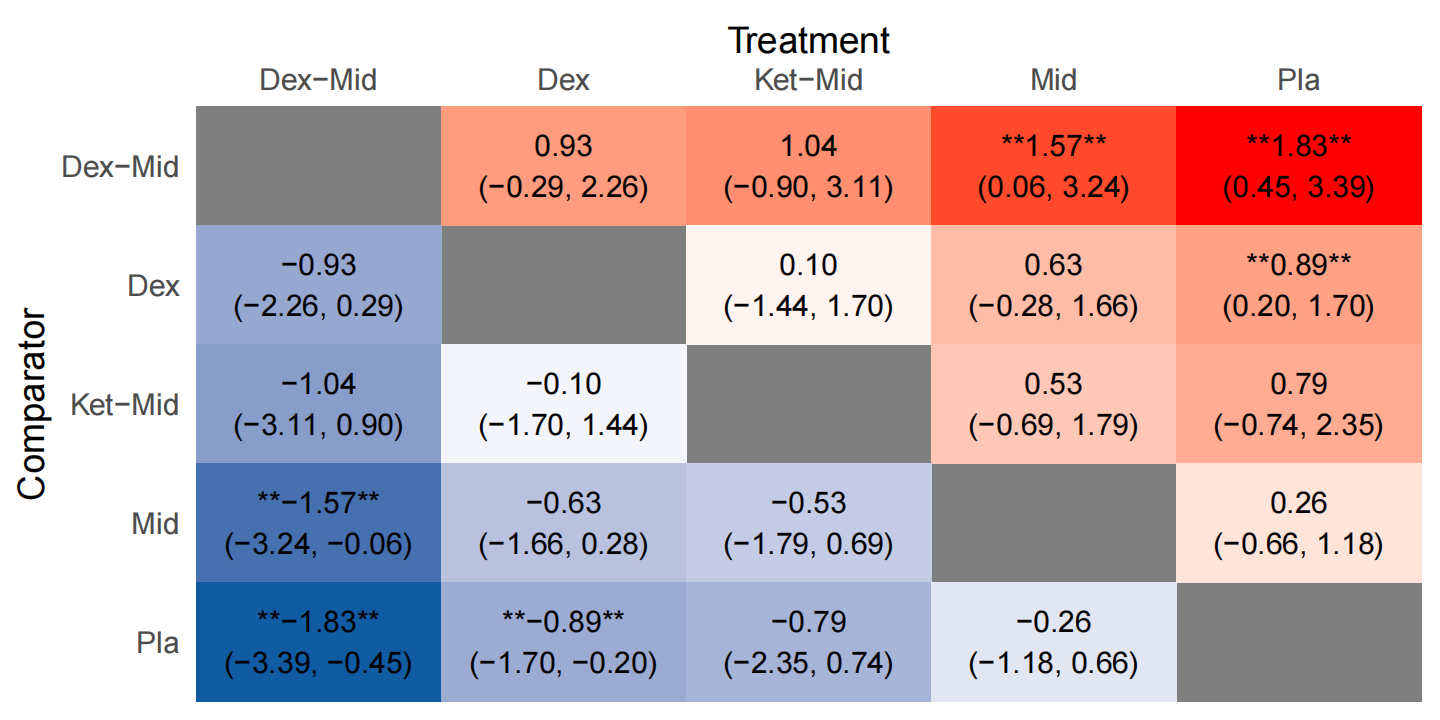


**Figure S6. League plots for dental/oral repair**


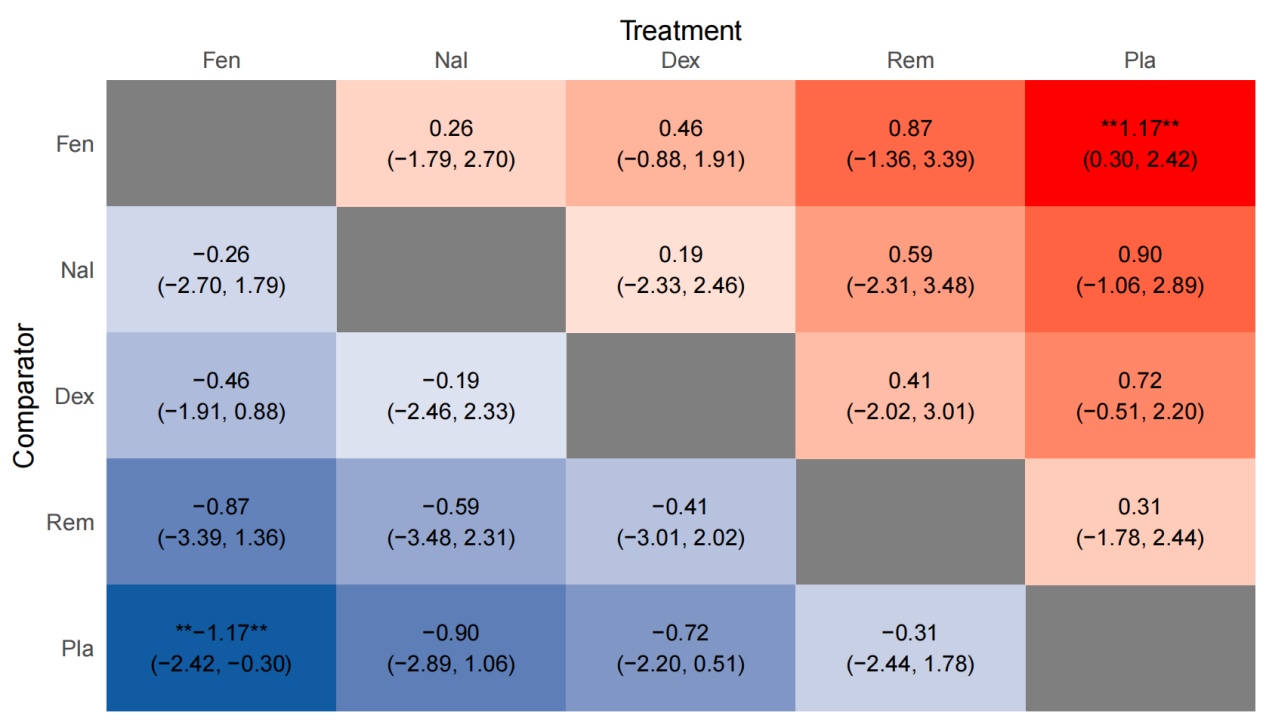


**Figure S7. League plots for myringotomy/cochlear implantation**
